# Supplementary material for: Synaptic Orb2A Bridges Memory Acquisition and Late Memory Consolidation in Drosophila
Source: Cell Rep. 2015 Jun 18;11(12):1953–65. doi: 10.1016/j.celrep.2015.05.037 (PMC4508346; doi:10.1016/j.celrep.2015.05.037)
Supplement: Document S1. Supplemental Experimental Procedures, Figures S1 and S2, and Tables S1–S12 [file mmc1.pdf]

Cell Reports

Supplemental Information

Synaptic Orb2A Bridges Memory Acquisition and Late Memory Consolidation in *Drosophila*

Sebastian Krüttner, Lisa Traunmüller, Ugur Dag, Katharina Jandrasits, Barbara Stepien, Nirmala Iyer, Lee G. Fradkin, Jasprina N. Noordermeer, Brett D. Mensh, and Krystyna Keleman

Supplemental Information

Figure S1 VT005526-LexA drives expression exclusively in the aSP13 dopaminergic neurons (Figure 2B and 2C)

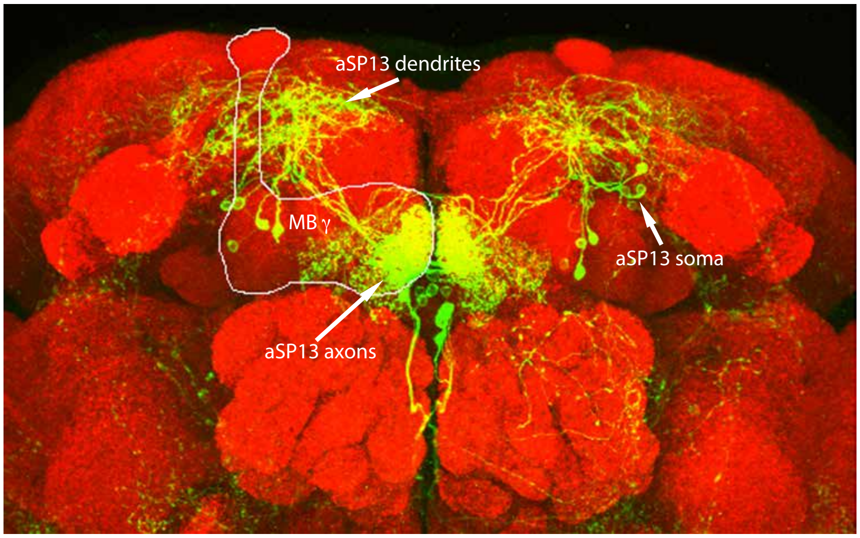

Expression pattern of the VT005526-LexA line used for the activation and silencing experiments with LexAop-TrpA1 and LexAop-shi^{ts} respectively, shown in the Figure 2B and 2C. There are typically 2-6 aSP13 neurons per hemisphere. The presynaptic termini of aSP13 neurons (axons) are located at the tip to the MB γ lobe. The postsynaptic termini (dendrites) are located in the medial protocerebrum.

Figure S2 On/Off kinetics of Orb2 in TARGET experiment (Figure 6)

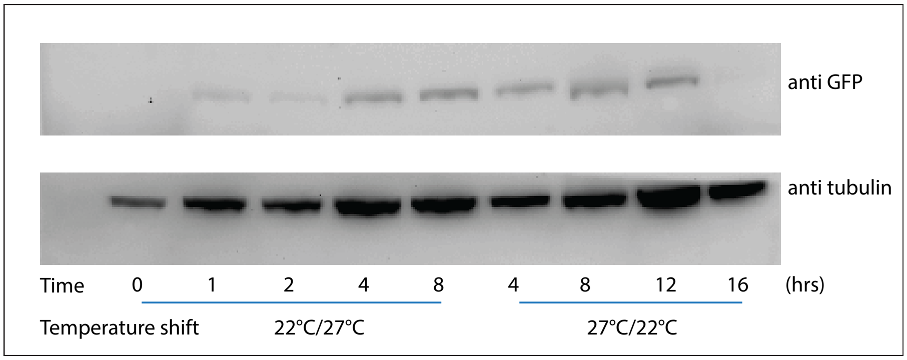

Head extracts from *w+;tubGal80ts, UAS-orb2BGFP;orb2*^{mCPEBRBD} adult flies were analyzed by IP and WB with Abs against the GFP tag at indicated time points after temperature shift either from 22°C to 27°C or back to 22°C after 7 hour induction at 27°C.

Table S1 Post-learning global activation of dopamine pathways (Figure 1)

	Genotype	Test	DA	Train	*n*	CI (%) mean±sd median	10%-ile 90%-ile	LI (%) mean±sem median	*P* *LI=0*	*P* $LI_n=LI_4$
1	*Canton-S*	20 min	-	-	57	52.3±26.3<br>50.0	15.00<br>90.00			
2	*Canton-S*	20 min	-	+	57	13.9±18.9<br>5.0	0.00<br>31.00	73.5±5.0<br>90.0	0.000<br>0.000	0.000<br>0.000
3	*Canton-S*	24 hrs	-	-	54	53.4±25.4<br>60.0	12.50<br>85.00			
4	*Canton-S*	24 hrs	-	+	61	51.1±25.3<br>55.0	15.00<br>85.00	4.4±8.7<br>8.3	0.319<br>0.473	
5	*Canton-S*	24 hrs	-	-	54	53.4±25.4<br>60.0	12.50<br>85.00			
6	*Canton-S*	24 hrs	+	-	66	57.1±29.7<br>65.0	13.50<br>91.50	-7.1±9.7<br>-8.3	0.770<br>0.878	0.386<br>0.486
7	*Canton-S*	24 hrs	+	-	66	57.2±29.7<br>65.0	13.50<br>91.50			
8	*Canton-S*	24 hrs	+	+	67	30.1±24.8<br>25.0	0.00<br>66.00	47.3±6.3<br>61.5	0.000<br>0.000	0.000<br>0.002
9	*Canton-S*	24 hrs<br>(w/ virgin)	+	-	45	79.3±28.4<br>90.0	31.00<br>100			
10	*Canton-S*)	24 hrs<br>(w/ virgin)	+	+	57	66.1±32.5<br>75.00	5.00<br>100	16.6±7.0<br>16.7	0.017<br>0.018	0.304<br>0.512

Courtship indices of the *n* Canton-S males fed with sucrose only (DA-) or supplemented with dopamine (DA+) after being trained with a mated female for 1 hr (Train +) or staying alone (Train-) as indicated in Fig. 1, and tested in single-pair assays with mated or virgin females when indicated. *P* values determined by permutation test for the null hypothesis that learning equals 0 (H_0: LI = 0) or for the null hypothesis that specific experimental and control males learn equally well (H_0: $LI_n = LI_c$)

Table S2 Post-learning thermogenetic activation of PAM-DA neurons (Figure 2A)

	Genotype	Time at 32˚C	Train	*n*	CI (%) mean±sd median	10%-ile 90%-ile	LI (%) mean±sem median	*P* *LI=0*	*P* $LI_n=LI_{18}$
1	*HL09-Gal4*, *UAS-TrpA1*, 32˚C	2-4	-	34	82.8±29.5<br>100.0	20.00<br>100			
2	*HL09-Gal4*, *UAS-TrpA1*, 32˚C	2-4	+	36	71.6±34.3<br>90.0	8.50<br>100	13.5±5.0<br>10.0	0.067<br>0.131	0.091<br>0.028
3	*HL09-Gal4*, *UAS-TrpA1*, 32˚C	4-6	-	34	82.5±27.5<br>95.0	29.00<br>100			
4	*HL09-Gal4*, *UAS-TrpA1*, 32˚C	4-6	+	36	71.4±30.1<br>75.0	25.00<br>100	13.5±8.0<br>21.05	0.052<br>0.064	0.073<br>0.028
5	*HL09-Gal4*, *UAS-TrpA1*, 32˚C	6-8	-	36	76.0±24.0<br>80.0	33.50<br>100			
6	*HL09-Gal4*, *UAS-TrpA1*, 32˚C	6-8	+	34	65.7±30.2<br>70.0	25.00<br>100	13.5±8.2<br>12.5	0.061<br>0.244	0.075<br>0.120
7	*HL09-Gal4*, *UAS-TrpA1*, 32˚C	8-10	-	35	83.1±21.9<br>95.0	52.50<br>100			
8	*HL09-Gal4*, *UAS-TrpA1*, 32˚C	8-10	+	36	44.2±33.5<br>40.0	5.00<br>97.00	46.9±7.2<br>57.9	0.000<br>0.000	0.000<br>0.000
9	*HL09-Gal4*, *UAS-TrpA1*, 32˚C	10-12	-	34	58.5±29.6<br>55.0	22.50<br>100			
10	*HL09-Gal4*, *UAS-TrpA1*, 32˚C	10-12	+	34	64.7±36.0<br>80.0	7.50<br>100	-10.6±14.2<br>-45.4	0.772<br>0.937	0.922<br>0.680

11	*HL09-Gal4, UAS-TrpA1*, 32˚C	12-14	-	36	59.3±27.9<br>60.00	23.50<br>96.50			
12	*HL09-Gal4, UAS-TrpA1*, 32˚C	12-14	+	36	59.0±33.2<br>55.00	13.50<br>100	0.7±5.0<br>8.3	0.485<br>0.434	0.478<br>0.491
13	*HL09-Gal4, UAS-TrpA1*, 32˚C	14-16	-	33	87.9±20.5<br>95.00	67.00<br>100			
14	*HL09-Gal4, UAS-TrpA1*, 32˚C	14-16	+	36	80.7±30.4<br>95.0	15.50<br>100	8.2±6.9<br>0.0	0.134<br>0.964	0.141<br>0.009
15	*HL09-Gal4, UAS-TrpA1*, 32˚C	16-18	-	33	92.6±13.1<br>95.0	77.00<br>100			
16	*HL09-Gal4, UAS-TrpA1*, 32˚C	16-18	+	36	87.9±22.2<br>95.0	61.00<br>100	5.0±4.6<br>0.0	0.149<br>0.970	0.166<br>0.001
17	*HL09-Gal4, UAS-TrpA1*, 22˚C	-	-	30	66.7±34.7<br>75.0	5.50<br>100			
18	*HL09-Gal4, UAS-TrpA1*, 22˚C	-	+	34	75.0±33.2<br>95.0	10.00<br>100	-12.5±13.7<br>-26.6	0.835<br>0.877	
19	*UAS-TRPA1/+*, 32˚C	8-10	-	35	54.9±33.1<br>55.0	10.00<br>95.00			
20	*UAS-TRPA1/+*, 32˚C	8-10	+	34	55.0±30.2<br>55.0	10.00<br>92.50	-0.2±13.9<br>0.0	0.511<br>0.636	0.533<br>0.572
21	*HL09-Gal4/+*, 32˚C	8-10	-	35	50.9±29.9<br>40.0	15.00<br>100			
22	*HL09-Gal4/+*, 32˚C	8-10	+	35	44.3±27.6<br>35.0	15.00<br>92.00	12.9±5.0<br>12.5	0.169<br>0.242	0.208<br>0.433
23	*HL09-Gal4, UAS-TrpA1*, 32˚C<br>+PSI	8-10	-	36	63.9±34.4<br>70.00	15.00<br>100			
24	*HL09-Gal4, UAS-TrpA1*, 32˚C<br>+PSI	8-10	+	34	66.8±29.1<br>70.0	17.50<br>100	-4.5±12.2<br>0.0	0.642<br>0.657	0.665<br>0.573

Courtship indices of males of the indicated genotypes, retained at 22˚C or warmed to 32˚C for 2 hrs at the time points according to Fig. 2A after either 1 hour training with a mated female (Train+) or staying alone (Train-) as indicated above and tested in single-pair assays with mated females. *P* values determined by permutation test for the null hypothesis that learning equals 0 (H_0: LI = 0) or for the null hypothesis that specific experimental and control males learn equally well (H_0: $LI_n = LI_c$)

Table S3 Post-learning thermogenetic activation of aSP13-DA neurons (Figure 2B)

	Genotype	Time at 32˚C	Train	n	CI (%)<br>mean±sd<br>median	10%-ile<br>90%-ile	LI (%)<br>mean±sem<br>median	P LI=0	P LI_n=LI_c
1	*TH-Gal4, FF, UAS-TrpA1*, 32˚C	8-10	-	36	67.4±27.2<br>77.5	20.00<br>96.50			
2	*TH-Gal4, FF, UAS-TrpA1*, 32˚C	8-10	+	36	45.8±28.1<br>40.0	8.50<br>90.00	32.0±5.0<br>46.7	0.001<br>0.006	0.000<br>0.097
3	*VT005526-LexA, LexAop-TrpA1*, 32˚C	8-10	-	28	79.8±18.4<br>85.0	48.00<br>100			
4	*VT005526-LexA, LexAop-TrpA1*, 32˚C	8-10	+	34	60.2±35.0<br>50.00	7.50<br>100	24.6±7.6<br>41.2	0.005<br>0.001	0.006<br>0.003
5	*VT005526-LexA, LexAop-TrpA1*, 22˚C	-	-	34	60.9±33.9<br>65.0	12.50<br>100			
6	*VT005526-LexA, LexAop-TrpA1*, 22˚C	-	+	34	71.0±25.1<br>77.5	27.50<br>100	-16.7±13.2<br>-25.0	0.915<br>0.862	
7	*VT005526-LexA/+, 32˚C*	8-10	-	17	82.9±13.4<br>85.0	64.00<br>105			
8	*VT005526-LexA/+, 32˚C*	8-10	+	16	80.3±20.1<br>87.5	46.00<br>100	3.2±7.1<br>0.0	0.356<br>0.891	0.272<br>0.269
9	*TH-Gal4*, 32˚C	8-10	-	36	55.0±23.1<br>45.0	30.00<br>95.00			
10	*TH-Gal4*, 32˚C	8-10	+	36	60.6±22.6	25.00	-10.1±10.3	0.857	0.771

					57.5	90.00	-22.2	0.906	0.407
11	*TH-Gal4, FF, UAS-TrpA1*, 22°C	-	-	33	63.9±25.1<br>75.0	25.00<br>95.00			
12	*TH-Gal4, FF, UAS-TrpA1*, 22°C	-	+	29	73.1±19.1<br>75.0	45.00<br>100	-14.3±5.0<br>0.0	0.944<br>0.769	

Courtship indices of males of the indicated genotypes, retained at 22°C or warmed to 32°C for 2 hrs according to Fig. 2B after either 1 hour training with a mated female (Train+) or staying alone (Train-) as indicated above and tested in single-pair assays with mated females. *P* values determined by permutation test for the null hypothesis that learning equals 0 (H_0: LI = 0) or for the null hypothesis that specific experimental and control males learn equally well (H_0: $LI_n = LI_c$)

Table S4 Post-learning thermogenetic silencing of aSP13-DA neurons (Figure 2C)

	**Genotype**	**Train**	***n***	**CI (%)**<br>mean±sd<br>median	**10%-ile**<br>**90%-ile**	**LI (%)**<br>mean±sem<br>median	***P***<br>***LI=0***	***P*** $LI_n=LI_6$
1	*VT005526-LexA, LexAop-*shi^{ts}, 22°C	-	33	88.3±18.0<br>95.0	62.00<br>100			
2	*VT005526-LexA, LexAop-*shi^{ts}, 22°C	+	36	58.2±26.5<br>62.5	12.00<br>91.50	34.1±5.0<br>36.8	0.000<br>0.000	
3	*VT005526-LexA, LexAop-*shi^{ts}, 32°C	-	36	92.5±18.5<br>100	76.00<br>100			
4	*VT005526-LexA, LexAop-*shi^{ts}, 32°C	+	33	85.0±17.5<br>90.0	60.00<br>100	8.1±4.5<br>10.0	0.052<br>0.023	0.001<br>0.001
5	*LexAop-*shi^{ts}, 32°C	-	35	68.7±25.3<br>75.0	33.00<br>95.00			
6	*LexAop-*shi^{ts}, 32°C	+	33	46.7±26.9<br>45.0	5.00<br>85.00	32.1±8.0<br>40.0	0.001<br>0.001	0.831<br>0.883
7	*VT005526-LexA*, 32°C	-	29	60.9±17.5<br>65.0	35.00<br>85.00			
8	*VT005526-LexA*, 32°C	+	33	41.9±23.4<br>45.0	4.00<br>73.00	31.1±7.6<br>30.8	0.000<br>0.009	0.767<br>0.718

Courtship indices of males of the indicated genotypes, retained at 22°C or warmed to 32°C according to Fig. 2C after 7 hour training with a mated female (Train+) or staying alone (Train-) as indicated above and tested in single-pair assays with mated females. *P* values determined by permutation test for the null hypothesis that learning equals 0 (H_0: LI = 0) or for the null hypothesis that specific experimental and control males learn equally well (H_0: $LI_n = LI_c$)

Table S5 Post-acquisition inactivation of DopR1 after training for LTM (Figure 3A)

	**Genotype**	**Train**	**n**	**CI (%)**<br>mean±sd<br>median	**10%-ile**<br>**90%-ile**	**LI (%)**<br>mean±sem<br>median	**P LI=0**	**P** $LI_n=LI_2$
1	*Canton-S*	-	59	78.7±21.5<br>85.0	45.00<br>100			
2	*Canton-S*	+	60	49.8±30.8<br>45.0	10.00<br>100	36.8±5.0<br>47.1	0.000<br>0.000	
3	$DopR1^{attp}$	-	54	78.7±22.8<br>85.0	42.50<br>100			
4	$DopR1^{attp}$	+	51	70.4±28.6<br>75.0	21.00<br>97.00	10.6±6.2<br>11.8	0.051<br>0.074	0.002<br>0.006
5	$DopR2^{attp}$	-	69	67.0±25.9<br>75.0	35.00<br>95.00			
6	$DopR2^{attp}$	+	67	41.3±32.2<br>30.0	5.00<br>91.00	38.4±6.5<br>60.0	0.000<br>0.000	0.857<br>0.381
7	$DopR1^{res}$	-	30	65.5±28.8<br>65.0	20.00<br>100			
8	$DopR1^{res}$	+	31	45.7±27.9	6.00	30.3±9.5	0.004	0.521

				45.0	89.00	30.8	0.051	0.142
9	*Canton-S*+ SCH23390	-	36	63.0±26.9<br>65.0	23.50<br>96.50			
10	*Canton-S*+ SCH23390	+	36	63.1±24.5<br>60.0	32.00<br>95.00	-0.4±9.7<br>7.7	0.519<br>0.402	0.000<br>0.003
11	*DopR2*^{attp}+ SCH23390	-	36	40.0±24.9<br>35.0	85.50<br>78.00			
12	*DopR2*^{attp}+ SCH23390	+	32	32.7±24.3<br>25.0	50.00<br>78.50	18.4±13.6<br>28.6	0.108<br>0.230	0.127<br>0.323<br>P LI_n=LI_{10}

Courtship indices of males of the indicated genotypes either trained for 7 hrs with a mated female (Train+) or remaining alone (Train-) as indicated in Fig. 3A and tested in single-pair assays with mated females. Indicated males were fed for 6 hrs with DopR1&2 antagonist, SCH23390, after 7 hrs training on water only. *P* values determined by permutation test for the null hypothesis that learning equals 0 (H_0: LI = 0) or for the null hypothesis that specific experimental and control males learn equally well (H_0: LI_n = LI_c)

Table S6 Post-acquisition inactivation of DopR1 (Figure 3B)

	Genotype	DA	Train	n	CI (%)<br>mean±sd<br>median	10%-ile<br>90%-ile	LI (%)<br>mean±sem<br>median	P LI=0	P LI_n=LI_2
1	*Canton-S*	+	-	72	63.4±29.4<br>75.0	16.50<br>98.50			
2	*Canton-S*	+	+	70	39.4±28.6<br>35.00	0.50<br>89.50	39.7±5.0<br>53.3	0.000<br>0.000	
3	*DopR1*^{attp}	+	-	36	70.9±27.7<br>75.0	27.50<br>100			
4	*DopR1*^{attp}	+	+	33	72.1±22.8<br>75.0	37.00<br>100	-1.6±8.7<br>0.0	0.572<br>0.632	0.000<br>0.001
5	*DopR2*^{attp}	+	-	53	58.6±27.9<br>60.0	15.00<br>95.00			
6	*DopR2*^{attp}	+	+	67	34.6±30.0<br>25.0	0.00<br>82.00	40.9±7.4<br>58.3	0.000<br>0.000	0.893<br>0.675
7	*Canton-S*	+<br>SCH23390	-	35	57.1±23.4<br>60.0	30.00<br>90.00			
8	*Canton-S*	+<br>SCH23390	+	35	58.0±23.9<br>65.0	23.00<br>87.00	-1.5±10.0<br>-8.3	0.561<br>0.762	0.001<br>0.002
9	*DopR2*^{attp}	+<br>SCH23390	-	36	44.4±31.0<br>40.0	1.50<br>85.00			
10	*DopR2*^{attp}	+<br>SCH23390	+	32	37.5±29.7<br>30.0	3.50<br>81.50	15.5±15.3<br>25.0	0.181<br>0.177	0.355<br>0.303<br>P LI_n=LI_8

Courtship indices of males of the indicated genotypes fed with sucrose supplemented with dopamine (DA+) after being starved for 16 hrs and trained with a mated female for 1 hr (Train+) or remaining alone (Train-) as indicated in Fig. 3B and tested in single-pair assays with mated females. Indicated males were fed with dopamine supplemented with DopR1&2 antagonist SCH23390. *P* values determined by permutation test for the null hypothesis that learning equals 0 (H_0: LI = 0) or for the null hypothesis that specific experimental and control males learn equally well (H_0: LI_n = LI_c)

Table S7 LTM rescue with DopR1 in subsets of MB neurons (Figure 3C)

	Genotype	Train	n	CI (%)<br>mean±sd<br>median	10%-ile<br>90%-ile	LI (%)<br>mean±sem<br>median	P LI=0	P<br>LI_n=LI_2
1	*UAS-DopR1; DopR1*^{attp}	-	31	82.0±23.4<br>90.0	55.00<br>100			
2	*UAS-DopR1; DopR1*^{attp}	+	33	77.1±22.0	42.50	6.0±5.0	0.177	

				85.0	97.50	5.6	0.263	
3	*UAS-DopR1;c739-Gal4; DopR1*^{attp}	-	29	53.7±25.4<br>60.0	15.00<br>85.00			
4	*UAS-DopR1;c739-Gal4; DopR1*^{attp}	+	34	54.0±30.3<br>50.0	10.00<br>90.00	-0.6±13.0<br>16.7	0.518<br>0.402	0.644<br>0.681
5	*UAS-DopR1;305-Gal4; DopR1*^{attp}	-	20	84.5±15.7<br>90.0	60.00<br>100			
6	*UAS-DopR1;305-Gal4; DopR1*^{attp}	+	19	84.5±14.9<br>90.0	55.00<br>100	0.03±5.8<br>0.0	0.519<br>0.553	0.501<br>0.711
7	*UAS-DopR1;Y201-Gal4; DopR1*^{attp}	-	34	74.5±21.9<br>80.0	41.00<br>95.00			
8	*UAS-DopR1;Y201-Gal4; DopR1*^{attp}	+	36	44.9±26.0<br>42.5	10.00<br>80.00	39.8±6.6<br>50.0	0.000<br>0.000	0.001<br>0.009
9	*c739-Gal4; DopR1*^{attp}	-	31	89.7±14.5<br>95.0	67.00<br>100			
10	*c739-Gal4; DopR1*^{attp}	+	33	86.9±17.9<br>80.0	60.00<br>100	3.1±4.2<br>0.00	0.244<br>0.922	0.692<br>0.347
11	*305-Gal4; DopR1*^{attp}	-	32	77.8±21.0<br>75.0	45.00<br>98.50			
12	*305-Gal4; DopR1*^{attp}	+	36	65.1±24.5<br>67.5	28.50<br>100	16.3±6.6<br>23.5	0.014<br>0.004	0.261<br>0.087
13	*201-Gal4; DopR1*^{attp}	-	34	82.2±18.7<br>87.5	57.50<br>100			
14	*201-Gal4; DopR1*^{attp}	+	34	81.8±19.1<br>90.0	55.00<br>100	0.5±5.6<br>-5.9	0.484<br>0.868	0.500<br>0.339

Courtship indices of males of the indicated genotypes either trained for 7 hrs with a mated female (Train+) or remaining alone (Train-) as indicated in Fig. 3C and tested in single-pair assays with mated females. *P* values determined by permutation test for the null hypothesis that learning equals 0 (H_0: LI = 0) or for the null hypothesis that specific experimental and control males learn equally well (H_0: $LI_n = LI_c$)

Table S8 *orb2* mutant in courtship memory consolidation assay (Figure 4A)

	**Genotype**	**Train**	**DA**	**n**	**CI (%)** mean±sd median	**10%-ile 90%-ile**	**LI (%)** mean±sem median	**P LI=0**	**P LI_n=LI_2**
1	*Canton-S*	-	+	36	58.5±32.1<br>55.0	10.00<br>100			
2	*Canton-S*	+	+	35	27.9±25.2<br>25.0	0.00<br>62.00	52.3±8.5<br>54.5	0.000<br>0.000	
3	*orb2*$^{orb2\Delta Q}$	-	+	36	76.0±23.0<br>80.0	33.50<br>100			
4	*orb2*$^{orb2\Delta Q}$	+	+	33	73.5±19.3<br>75.0	45.00<br>98.00	3.2±6.8<br>6.2	0.330<br>0.209	0.000<br>0.022

Courtship indices of males of the indicated genotype fed with sucrose supplemented with dopamine (+ DA) after being trained with a mated female for 1 hr (Train+) or remaining alone (Train-) as indicated in Fig. 4A and tested in single-pair assays with mated females. *P* values determined by permutation test for the null hypothesis that learning equals 0 (H_0: LI = 0) or for the null hypothesis that specific experimental and control males learn equally well (H_0: $LI_n = LI_c$)

Table S9 Quantification of the Orb2 oligomers (Figure 4C)

Genotype	***Wt (-DA)***	***DopR1*$^{att\ p}$*(-DA)***	***DopR2*^{attp} *(-DA)***	***Wt (+DA)***	***DopR1*^{att} *(+DA)***	***DopR2*^{attp} *(+DA)***
Mean Intensity	358.83	316.12	229.34	6523.18	204.30	5546.77
MI_N /MI_{wt-DA}	1	0.88	0.64	18.18	0.79	15.46

WB signal corresponding to the Orb2-GFP oligomers (Fig. 4B) has been quantified using Fiji-ImageJ (Fig. 4C). Mean intensity was normalized to the wild type not treated with dopamine (wt - DA).

Table S10 Temporal rescue of LTM with Orb2A and Orb2B isoforms in MB (Figure 6)

	**Genotype**	***n***	**CI naïve (%)**<br>mean±sd<br>median	**10%-ile**<br>**90%-ile**	***n***	**CI exp (%)**<br>mean±sd<br>median	**10%-ile**<br>**90%-ile**	**LI (%)**<br>mean±sem<br>median	***P***<br>***LI=0***	***P***<br>$LI_{wt}=LI_{*}$
1	*TubG80^{ts};orb2$^{\Delta Q\Delta A}$;UAS-*<br>*Orb2A;MB247-Gal4*	34	26.8±29.5<br>10.0	0<br>77.50	36	24.9±21.8<br>15.0	0.00<br>58.00	7.1±5.0<br>-50.0	0.382<br>0.783	
1	*TubG80ts; orb2$^{\Delta Q\Delta A}$;UAS-*<br>*Orb2AΔQ;MB247-Gal4*	34	20.4±19.2<br>15.0	0<br>46.5	36	23.1±19.5<br>20.0	0.00<br>46.5	-12.8±2.3<br>-33.3	0.716<br>0.852	0.545<br>0.931
2	*TubG80ts; orb2$^{\Delta Q\Delta A}$;UAS-*<br>*Orb2A;MB247-Gal4*	35	71.4±17.5<br>75.0	45.00<br>90.00	36	48.6±28.9<br>45.0	3.50<br>81.50	31.9±7.2<br>40.0	0.000<br>0.000	
2	*TubG80ts; orb2$^{\Delta Q\Delta A}$;UAS-*<br>*Orb2AΔQ;MB247- Gal4*	33	62.1±27.6<br>70.00	50.00<br>95.00	43	57.2±28.2<br>65.0	10.00<br>95.00	7.9±9.9<br>7.1	0.222<br>0.499	0.039<br>0.109
3	*TubG80ts; orb2$^{\Delta Q\Delta A}$;UAS-*<br>*Orb2A;MB247-Gal4*	33	48.3±27.4<br>45.00	12.00<br>91.00	34	31.6±28.8<br>25.0	0.00<br>80.00	34.6±12.3<br>44.4	0.008<br>0.015	
3	*TubG80ts; orb2$^{\Delta Q\Delta A}$;UAS-*<br>*Orb2AΔQ;MB247-Gal4*	39	54.1±26.2<br>60.0	9.00<br>85.55	31	54.2±26.1<br>60.0	10.01<br>0.00	-0.2±5.0<br>0.0	0.498<br>0.571	0.046<br>0.049
4	*TubG80ts; orb2$^{\Delta Q\Delta A}$;UAS-*<br>*Orb2A;MB247-Gal4*	31	68.4±28.8<br>75.0	10.00<br>95.00	36	72.5±18.8<br>75.0	42.00<br>95.00	-6.0±9.1<br>0.0	0.760<br>0.843	
4	*TubG80ts; orb2$^{\Delta Q\Delta A}$;UAS-*<br>*Orb2AΔQ;MB247-Gal4*	36	67.6±27.5<br>75.0	20.50<br>95.00	34	66.9±24.5<br>70.0	27.50<br>95.00	1.1±9.0<br>6.7	0.463<br>0.421	0.578<br>0.744
5	*TubG80ts; orb2$^{\Delta Q\Delta A}$;UAS-*<br>*Orb2A;MB247-Gal4*	33	74.4±20.9<br>85.0	35.00<br>95.00	36	66.1±23.0<br>70.0	28.50<br>91.50	11.1±6.8<br>17.6	0.062<br>0.026	
5	*TubG80ts; orb2$^{\Delta Q\Delta A}$;UAS-*<br>*Orb2AΔQ;MB247-Gal4*	34	77.7±22.8<br>85.0	57.50<br>100	34	75.1±27.9<br>85.0	27.50<br>100	3.2±7.6<br>0.0	0.343<br>0.674	0.442<br>0.102
1’	*TubG80ts;orb2^{mCPEB2RBD};UAS*<br>*-Orb2B;MB247-Gal4*	32	57.0±30.7<br>60.0	15.00<br>95.00	36	52.9±28.5<br>45.0	13.50<br>95.00	7.2±5.0<br>25.0	0.285<br>0.245	
1’	*TubG80ts;orb2^{mCPEB2RBD};UAS*<br>*-Orb2BRBD*;MB247-Gal4*	18	54.2±25.8<br>50.0	19.50<br>95.50	18	45.6±17.8<br>45.0	9.50<br>76.00	15.9±13.0<br>10.0	0.136<br>0.374	0.650<br>0.645
2’	*TubG80ts;orb2^{mCPEB2RBD};UAS*<br>*-Orb2B;MB247-Gal4*	35	79.9±21.9<br>85.0	45.00<br>100	34	57.2±27.1<br>65.0	7.50<br>85.00	28.4±6.8<br>23.52	0.000<br>0.000	
2’	*TubG80ts;orb2^{mCPEB2RBD};UAS*<br>*-Orb2BRBD*;MB247-Gal4*	33	81.52±15.93<br>85.0	62.50<br>97.50	34	75.6±21.6<br>80.0	45.00<br>97.50	7.27±5.5<br>5.9	0.111<br>0.890	0.021<br>0.033
3’	*TubG80ts;orb2^{mCPEB2RBD};UAS*<br>*-Orb2B;MB247-Gal4*	35	66.6±22.5<br>70.0	30.00<br>92.00	34	62.7±26.9<br>65.0	23.00<br>95.00	5.9±8.6<br>7.1	0.263<br>0.550	
3’	*TubG80ts;orb2^{mCPEB2RBD};UAS*<br>*-Orb2BRBD*;MB247-Gal4*	26	52.9±33.0<br>60.0	3.00<br>95.00	27	46.9±33.7<br>40.0	5.00<br>91.00	11.4±5.0<br>33.3	0.273<br>0.134	0.747<br>0.268
4’	*TubG80ts;orb2^{mCPEB2RBD};UAS*<br>*-Orb2B;MB247- Gal4*	36	69.7±26.9<br>80.0	27.00<br>95.00	36	67.8±21.6<br>65.0	33.50<br>95.00	2.8±8.1<br>18.8	0.365<br>0.096	
4’	*TubG80ts;orb2^{mCPEB2RBD};UAS*<br>*-Orb2BRBD*;MB247-Gal4*	32	63.9±25.4<br>75.0	19.50<br>93.50	36	61.3±26.6<br>65.0	18.50<br>95.00	4.2±9.6<br>13.3	0.336<br>0.195	0.911<br>0.721
5’	*TubG80ts;orb2^{mCPEB2RBD};UAS*<br>*-Orb2B;MB247-Gal4*	36	79.0±23.3<br>85.0	41.00<br>100	36	57.5±28.1<br>65.0	5.00<br>85.00	27.2±7.1<br>23.5	0.000<br>0.007	
5’	*TubG80ts;orb2^{mCPEB2RBD};UAS*<br>*-Orb2BRBD*;MB247-Gal4*	25	79.6±24.2<br>90.0	38.00<br>97.00	32	73.3±26.8<br>85.0	31.00<br>100	7.9±8.1<br>5.6	0.188<br>0.277	0.074<br>0.266

Courtship indices of males of the indicated genotypes either trained for 7 hrs with a mated female (CI exp) or remaining alone (CI naive), treated as indicated in Fig. 6 and tested in single-pair assays with mated females. *P* values determined by permutation test for the null hypothesis that learning equals 0 (H_0: LI = 0) or for the null hypothesis that rescue flies with the wild type isoform learns equally well as rescue flies with the mutated isoform in the same conditions (H_0: LI_n = LI_*)

Table S11 Orb2 regulates translation of CaMKII (Figure 7A)

3'UTR	Npc2a-3'UTR-RA (control)			CaMKII-3'UTR-RH		
**Genotype**	**Orb2 wt**	**Orb2RRM***	**Ratio wt/RRM***	**Orb2 wt**	**Orb2RRM***	**Ratio wt/RRM***
FLuc/Rluc	16.183	14.680	1.1023	11.995	12.896	0.931
	16.272	15.319	1.0622	12.388	12.413	0.997
	45.179	43.740	1.0329	23.614	26.492	0.891
	42.370	44.147	0.9597	46.313	52.440	0.883
	6.380	4.599	1.3992	29.167	29.608	0.985
	7.629	7.367	1.0355	30.007	33.264	0.902
				14.852	21.392	0.694
Mean			1.098			0.896
SEM			0.0630			0.0164
P (T-test)			0.0225			

Dual luciferase reporter assay is S2 cells co-expressing either Firefly luciferase tethered to the CaMKII 3'UTR or control Npc2a-3'UTR (does not contain Orb2 specific binding sequence) and Renilla luciferase tethered to the SV40 3'UTR (Fig. 7A). The values represent Firefly luciferase signal normalized to Renilla luciferase fluorescence in S2 cells expressing either Orb2 wt or Orb2 with the RBD mutated, Orb2RRM*.

Table S12 Mean intensity of the *EYFP-CaMKII-3'UTR* in the MB gamma neurons (Figure 7C)

	Genotype	DA (hrs)	n	Mean intensity	SEM	P(ftx=ft0)	P (ftx=ft24)
1	+ *CamKII 3'UTR, wt Orb2*	0	4	28.21	5.5		0.99
2	+ *CamKII 3'UTR, wt Orb2*	6	3	50.50	4.8	0.03	0.04
3	+ *CamKII 3'UTR, wt Orb2*	12	6	50.63	4.9	0.02	0.03
4	+ *CamKII 3'UTR, wt Orb2*	24	3	28.23	6.2	0.99	

	Genotype	DA (hrs)	*n*	Mean intensity	SEM	*P*(ftx=ft0)
1	+ *CamKII 3'UTR*, $Orb2^{\Delta A}$,	0	6	21.96	4.3	
2	+ *CamKII 3'UTR*, $Orb2^{\Delta A}$	6	4	29.92	6.89	0.33

Medium intensity of the fluorescence measured in the gamma lobe of the MB of the indicated genotype according to Fig. 7C. *P* values determined by 2-sided t-test for the null hypothesis that the fluorescence intensity at time xhr equals the intensity at 0hr (H_0: ftx=ft0) or 24 hrs (H_0: ftx=ft24)

Material and methods

Courtship Conditioning Paradigm

Flies were maintained on conventional cornmeal-agar medium under a 12 hrs light: dark cycle at 25°C and 60% relative humidity. Courtship assays were performed at variable circadian clock of the flies. Males were assayed for courtship conditioning as described (Siwicki and Ladewski, 2003). For training, individual males were placed in food chambers either with (trained) or without (naive) a single premated female. After training, each male was recovered, transferred to a fresh food vial and kept in isolation until testing. For long-term memory, males were trained for 6–7 hrs and tested after 24 hrs. For short-term memory, the training period was 1 hr and the test was performed after 30 min. Tests were performed in a 10 mm diameter courtship chamber and

videotaped for 10 min (JVC handycam, 30 GB HD). Videos were scored manually and blind to the genotype for CI, which is the percentage of time each male spent courting during the test. Courtship index (CI) was used to calculate the Learning Index (LI): CI_{naive}-$CI_{trained}$/CI_{naive} × 100.

Immunohistochemistry

Immunohistochemistry on adult brains was performed as described (Yu et al., 2010). Fly brains were dissected (between 5 to 8 days after eclosion) in PBS and fixed using 4% paraformaldehyde in PBST (PBS with 0.3% Triton X-100) for 20 min at 24°C. After washing in PBST, the tissue was blocked in 5% normal goat serum in PBST for at least 2 hrs. The primary antibody and secondary antibody were incubated for 48 hrs at 4°C. The brains were washed with PBST 3 × 10 min and then overnight at 4°C between the primary and secondary antibody incubations. After the secondary antibody incubation, samples were washed 3 × 10 min and overnight at 4°C before mounting in Vectashield (VectorLabs). Antibodies used: rabbit polyclonal anti-GFP (1:5,000, Torri Pines); secondary Alexa-488 antibodies (1:1,000, Invitrogen).

Confocal Microscopy

For imaging and measurement of the fluorescence intensity of the EYFP+/- CaMKII-3'UTR, the fly brains immunostained as described above, were scanned using a Zeiss LSM 710 with a Zeiss Multi Immersion Plan NeoFluar 63× objective. Scanning parameters were set to image the entire mushroom body. Images were taken at 785 × 785 pixels. Images were processed in Imaris for fluorescence quantification. Briefly a cuboid of similar size was set as surface into each MB gamma lobe and the mean YFP fluorescence quantified.

Immunoprecipitation and Western Blot

Adult heads of the indicated genotype were lysed in homogenization buffer (PBS,150mM NaCl, 0.1mM CaCl2, 3mM MgCl2, 5% Glycerol, 1mM DTT, 0.1% TritonX100, 0.1% NP40, EDTA free protease inhibitor cocktail from Roche). The lysate was cleared by centrifugation prior to incubation with Chromotek GFPtrap beads (according to the manufacturer protocol). The proteins were transferred to a PVDF membrane (Millipore) overnight in the cold room at 35mv. Membrane was blocked in 5% milk prior to incubation for 1 hr with a primary antibody. After 3 washes in PBST (PBS+ 0,05%Tween20) membrane was incubated for 1 hr in a secondary antibody. The membrane was developed using SuperSignal West Femto Maximum Sensitivity Substrate (ThermoScientific). Antibodies used: anti-GFP (Abcam 6556 rabbit polyclonal, 1:2,000).

Immuno-EM on adult brains

The brains of 6-7 day old adult flies were dissected in cold fly saline and fixed for 3 hours on ice with 0.1% glutaraldehyde/4% formaldehyde in 0.07M sodium phosphate buffer, pH 7.4, rinsed with 0.1 M phosphate buffer containing 0.1% saponin, and incubated overnight with an HRP-conjugated rabbit anti-GFP polyclonal antibody (Life Technologies, A10260, anti-GFP, rabbit IgG fraction, horseradish peroxidase conjugate) at 1:200 dilution in 0.1 M phosphate buffer containing 5% normal goat serum/1% BSA at 4° C. The brains were then rinsed with 0.1 M phosphate/0.1% saponin buffer and reacted with 0.5 mg/ml DAB in 0.1 M phosphate buffer containing 0.1% saponin for 45 minutes following the addition of 10 μl 0.03% H_2O_2. The brains were then rinsed with 0.1 M Na-cacodylate buffer, followed by OTOT enhancement (10 min cycles of 0.01% OsO4 in 0.1M Na-cacodylate buffer followed by 0.1% thiocarbohydrazid1M Na-

cacodylate buffer with 0.1M Na-cacodylate buffer rinses between steps), and a final 1% OsO_4 in 0.1 M Na-cacodylate buffer step for 1 hour at room temperature. Following osmication, the samples were rinsed with 0.1 M Na-cacodylate buffer, water and then dehydrated in ethanol followed by propylene oxide and embedded in Eponate 12 resin (Ted Pella, Redding, CA). Embedded brains were imaged using a Zeiss Versa 510 X-ray microscope operated at 40kV and 0.7 mm/pixel resolution. The computed tomograms were used to provide coordinates of the cell bodies of the Kenyon cells and gamma lobes of the mushroom body in each sample. A Leica Ultracut 6 ultramicrotome was used to cut 90 nm sections at the level of the Kenyon cells somata and gamma lobes of the mushroom body. Unstained sections were imaged with an FEI Spirit BioTWIN TEM operated at 80kV.

CaMKII translation suppression assay

Luciferase reporter assay was done essentially as described (Mastushita-Sakai et al., 2010). In short, Orb2 plasmids were prepared by amplifying Orb2 CDS using primers containing attB sites. For Orb2 RRM1&2* site directed mutagenesis was used. All primers are listed below. Products were cloned into pDONR221 and recombined to obtain pAWM-Orb2B WT or RRM1&2* vectors. To create control (*Renilla*) and test (Firefly) luciferase expressing constructs pAMW (The Drosophila Gateway Vector Collection) was cut with BamHI (Fermentas) and the backbone fragment was ligated with MCS (multiple cloning site). Next, the Gateway expression cassette was PCR amplified from pAMW with casAWMf/casAWMr primers and cloned into the pAMW-MCS linearised with NheI/Asp718 (Roche) to obtain pAMW-cassette. For pAMW-Fluc-cassette destination vector the Firefly luciferase PCR product obtained by amplifying pAC-Fluc-6xBS with Flucf/Flucr primers was digested with SpeI/NheI and cloned into NheI cut pAMW-cassette vector. For pAMW-Rluc-cassette-polyA first the polyA signal sequence was amplified from pAMW with polAf/polAr primers and cloned with Asp718/XhoI. Subsequently, *Renilla* luciferase was PCR amplified from pAC-Rluc with Rlucf/Rlucr primers and cloned SpeI/NheI into NheI cut vector. To create final pAMW-Rluc-SV403'UTR-polyA vector SV40 3'UTR was PCR amplified from pAMW with SV403UTRf/SV403UTRr primers and cloned first into pDONR221 and then recombined with pAMW-Rluc-cassette-polyA. CaMKII 3'UTR and Npc2a-RA 3'UTR were PCR amplified using the following primers containing attB sites. PCR product was sub-cloned into pDONR221 vector using Gateway technology (Life Technologies). Resulting entry clones were recombined with pAMW-Fluc-cassette to obtain pAMW-Fluc-3'UTR plasmids. All constructs were confirmed by sequencing.

S2 cells were grown in semi-adhering liquid cultures at 27°C in water-jacketed incubator, with 5% CO_2 in liquid Schneider's Drosophila Medium (Invitrogen) supplemented with 10% fetal calf serum and PenStrep (Invitrogen) without agitation. S2 cells were split 1:10, grown overnight and diluted in Schneider's Drosophila Medium to 1mln/ml. Cells were transferred to 96-well culture plates 100µl per well. 120ng of DNA was used per transfection containing 10ng of pAMW-Rluc-SV40-polyA, 10ng of pAMW-Fluc-3'UTR reporter plasmid and 5ng pAWM-protein filled up to final DNA amount by inert bacterial plasmid pGEX-2T (GE Healthcare). Cells were transfected by adding 4.7µl of DNA/FuGENE (Promega FuGENE® HD Transfection Reagent) mix per 100ul cells and pipetting up and down. After 48h cells were transferred to deep well plates in 600µl 1xPBS, harvested by spinning 5 minutes at 800g, washed twice with 400µl 1xPBS and lysed in 30µl 1xPassive Lysis Buffer (Dual-Luciferase Reporter Assay System, Promega). For dual luciferase assay 10µl of lysate was pipetted onto 96-well plate and luciferase signals were measured in Synergy Plate Reader (BioTek) by adding 20µl Luciferase Assay Substrate in Luciferase Assay Buffer II, shaking, incubating 2 minutes and measure and 20µl Stop & Glo Substrate in Stop & Glo Buffer, shaking, incubating 2 minutes and measuring. Firefly luciferase signal was normalised to *Renilla* luciferase and ratio between signal from cells expressing Orb2B WT to Orb2B RRM1&2* mutant protein was calculated from the means for 3 replicates from the

same transfection. Each reaction was repeated in at least 6 independent experiments. Mean ratios between WT and mutant protein signals were calculated and compared to negative control (Npc2a-RA 3'UTR) using unpaired Student *t* test.

Mastushita-Sakai, T., White-Grindley, E., Samuelson, J., Seidel, C., and Si, K. (2010). Drosophila Orb2 targets genes involved in neuronal growth, synapse formation, and protein turnover. Proc Natl Acad Sci U S A *107*, 11987-11992.

Siwicki, K.K., and Ladewski, L. (2003). Associative learning and memory in Drosophila: beyond olfactory conditioning. Behav Processes *64*, 225-238.

Yu, J.Y., Kanai, M.I., Demir, E., Jefferis, G.S., and Dickson, B.J. (2010). Cellular organization of the neural circuit that drives Drosophila courtship behavior. Curr Biol *20*, 1602-1614.
