# Supplementary material for: Synaptic Orb2A Bridges Memory Acquisition and Late Memory Consolidation in Drosophila
Source: Cell Rep. 2015 Jun 18;11(12):1953–65. doi: 10.1016/j.celrep.2015.05.037 (PMC4508346; doi:10.1016/j.celrep.2015.05.037)
Supplement: Document S2. Article plus Supplemental Information [file mmc2.pdf]

Cell Reports

Article

Synaptic Orb2A Bridges Memory Acquisition and Late Memory Consolidation in *Drosophila*

Graphical Abstract

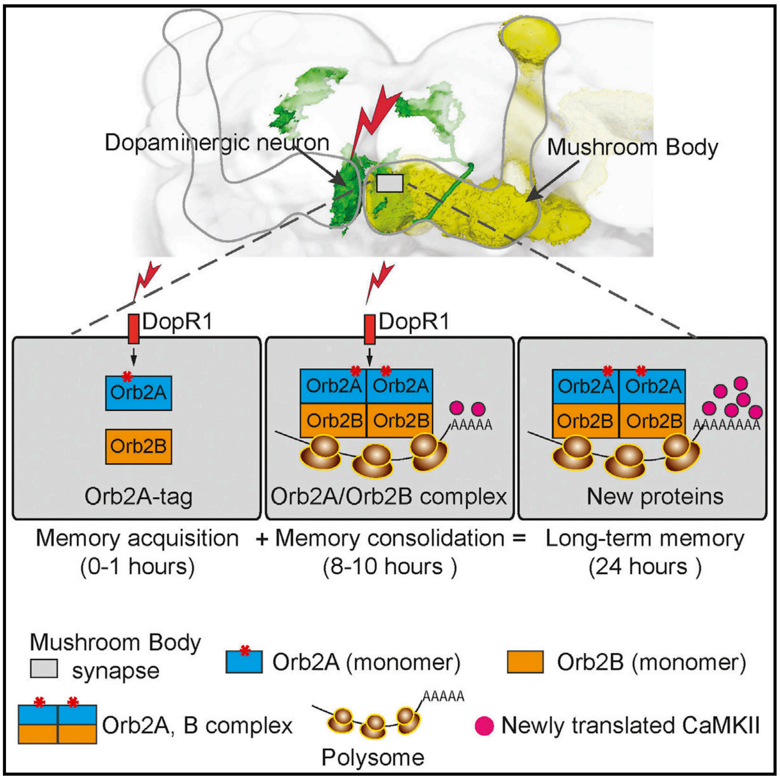

Authors

Sebastian Krüttner, Lisa Traunmüller, Ugur Dag, ..., Jasprina N. Noordermeer, Brett D. Mensh, Krystyna Keleman

Correspondence

kelemank@janelia.hhmi.org

In Brief

Krüttner et al. demonstrate that, similar to mammals, invertebrates exhibit late memory consolidation mediated by neural pathways engaged earlier during memory acquisition. Late reactivation of a dopaminergic neuron class in *Drosophila* recruits Orb2B to form complexes in synapses tagged by Orb2A upon memory acquisition; these complexes regulate CaMKII translation.

Highlights

- *Drosophila* exhibits late long-term memory consolidation
- DA-aSP13 neurons mediate both memory acquisition and memory consolidation
- *Drosophila* CPEB, Orb2, acts downstream of DopR1 in the MB γ neurons
- Synaptic Orb2A is required during memory acquisition and Orb2B during consolidation

Krüttner et al., 2015, Cell Reports *11*, 1953–1965
June 30, 2015 ©2015 The Authors
http://dx.doi.org/10.1016/j.celrep.2015.05.037

Cell Reports
Article

OPEN ACCESS
CellPress

Synaptic Orb2A Bridges Memory Acquisition and Late Memory Consolidation in *Drosophila*

Sebastian Krüttner,[2,3,7] Lisa Traunmüller,[2,4,7] Ugur Dag,[1,7] Katharina Jandrasits,[2] Barbara Stepien,[2,5] Nirmala Iyer,[1] Lee G. Fradkin,[6] Jasprina N. Noordermeer,[6] Brett D. Mensh,[1] and Krystyna Keleman[1,2,*]
[1]Janelia Research Campus, HHMI, 19700 Helix Drive, Ashburn, VA 20147, USA
[2]Research Institute of Molecular Pathology, Doktor Bohr Gasse 7, A-1030 Vienna, Austria
[3]Friedrich Miescher Institute for Biomedical Research, Maulbeerstrasse 66, 4058 Basel, Switzerland
[4]Biozentrum, University of Basel, Klingelbergstrasse 50-70, 4056 Basel, Switzerland
[5]The Max Planck Institute of Molecular Cell Biology and Genetics, Pfotenhauerstrasse 108, 01307 Dresden, Germany
[6]Department of Molecular Cell Biology, Leiden University Medical Center, 2300 RA Leiden, the Netherlands
[7]Co-first author
*Correspondence: kelemank@janelia.hhmi.org
http://dx.doi.org/10.1016/j.celrep.2015.05.037
This is an open access article under the CC BY license (http://creativecommons.org/licenses/by/4.0/).

SUMMARY

To adapt to an ever-changing environment, animals consolidate some, but not all, learning experiences to long-term memory. In mammals, long-term memory consolidation often involves neural pathway reactivation hours after memory acquisition. It is not known whether this delayed-reactivation schema is common across the animal kingdom or how information is stored during the delay period. Here, we show that, during courtship suppression learning, *Drosophila* exhibits delayed long-term memory consolidation. We also show that the same class of dopaminergic neurons engaged earlier in memory acquisition is also both necessary and sufficient for delayed long-term memory consolidation. Furthermore, we present evidence that, during learning, the translational regulator Orb2A tags specific synapses of mushroom body neurons for later consolidation. Consolidation involves the subsequent recruitment of Orb2B and the activity-dependent synthesis of CaMKII. Thus, our results provide evidence for the role of a neuromodulated, synapse-restricted molecule bridging memory acquisition and long-term memory consolidation in a learning animal.

INTRODUCTION

The brain rapidly learns environmental associations and behavioral contingencies but is selective about which lessons it commits to long-term memory. Evidence from multiple approaches has provided clues to how something learned becomes something remembered as well as how and where memories are stored. Neuromodulatory systems are thought to exert substantial control over whether a learning experience is memorable. Neuromodulatory pathways are activated during and/or after "important" behavioral moments for memory acquisition and memory consolidation (Plaçais et al., 2012; Rossato et al., 2009; Schwaerzel et al., 2003; Wise, 2004), and blocking them inhibits the formation of memories (Schwaerzel et al., 2003; Wise, 2004). Intriguingly, the process of memory consolidation often involves a critical period, sometimes many hours after the initial learning period, during which a reactivation of brain activity is required. In some cases, this reactivation involves a literal replay of a learning experience, either during the awake (Carr et al., 2011) or sleeping (Wilson and McNaughton, 1994) states.

Adult *Drosophila* flies exhibit remarkable behavioral complexity that can be modified by experience. They can learn to avoid or approach odors that were previously associated with an electric shock (Quinn et al., 1974) or with sugar reward (Tempel et al., 1984), respectively. Flies can also learn visual, tactile, and even spatial cues (Guo et al., 1996; Ofstad et al., 2011; Wustmann et al., 1996). A robust form of memory in flies is courtship conditioning, whereby naive males learn to preferentially court receptive virgin females after experiencing unsuccessful courtship of already mated (and therefore unreceptive) females. Depending on the learning experience, flies can form memories lasting from minutes to hours to several days (McBride et al., 1999; Siegel and Hall, 1979). However, the mechanisms that trigger production of long-term memory are not clear. Recently, it has been found that orchestrated activity of three clusters of dopaminergic neurons positively affect long-term memory formation during olfactory learning (Plaçais et al., 2012) and delayed activity of the specific dopaminergic neurons is critical for consolidation of the long-term appetitive memory (Musso et al., 2015).

Short-term memory can be mediated by a variety of protein-synthesis-independent mechanisms (Kandel, 2001). Long-term memories are thought to reflect protein-synthesis-dependent morphological and biochemical changes taking place in specific synapses within neuronal networks (Sutton and Schuman, 2006). Because more synapses in the brain are activated during memory acquisition than eventually might become consolidated, there must be mechanisms for determining which particular synapses will ultimately encode a given long-term memory.

CrossMark

Furthermore, these mechanisms must be capable of maintaining such specificity during the interval between memory acquisition and its consolidation.

The synaptic tag and capture hypothesis (Frey and Morris, 1997; Martin et al., 2000) proposes how specific synapses come to store a given memory. The original experimental evidence in support of this hypothesis came from in vitro electrophysiological studies in hippocampal slices. Conversion of early long-term potentiation (E-LTP, an in vitro correlate of short-term memory) into L-LTP (a physiological correlate of long-term memory) at synapses activated by a strong stimulation after a weak one suggested that synapses activated during behavioral memory acquisition might be tagged for a protein-synthesis-dependent long-term consolidation. Behavioral studies in rodents demonstrated that training that elicits short-term memory can be consolidated into long-term memory by a novel experience capable of inducing protein synthesis (Moncada and Viola, 2007). Moreover, activation of the dopaminergic ventral tegmental area in rats after learning induces protein synthesis, which is required for long-term memory consolidation (Rossato et al., 2009). Nonetheless, the molecular mechanisms of synaptic tagging have not been identified within the contexts of specific neuronal pathways and learning animals.

Candidates for such a synaptic tag are members of the cytoplasmic polyadenylation element binding family of proteins (CPEB) (Si et al., 2003a). CPEB proteins can be divided into two subfamilies. The CPEB-I subfamily includes the *Xenopus* CPEB1 and its *Drosophila* ortholog Orb1, which both regulate mRNA translation during oogenesis (Mendez and Richter, 2001). Members of the CPEB-II subfamily have been found to function in synaptic plasticity (mCPEB2–4; Richter, 2001) or long-term memory formation (*Drosophila* Orb2; Keleman et al., 2007; Majumdar et al., 2012). Almost all CPEBs exist in multiple variants generated by alternative mRNA splicing (Theis et al., 2003; Wang and Cooper, 2009). Orb2 has two isoforms, which contain a glutamine-rich domain (Q domain), present also in some, but not all, CPEBs in other species (Hafer et al., 2011; Si et al., 2003a). Orb2A recruits Orb2B into complexes, essential for memory persistence, through its Q domain upon neuronal activation. After being recruited into complexes, Orb2B regulates translation through its RNA-binding domain (Krüttner et al., 2012; Majumdar et al., 2012). A protein network has been recently identified that links neuronal activity and the reactivity of Orb2A (White-Grindley et al., 2014). Together, these data suggest that Orb2 and its CPEB homologs are promising candidates to serve as a molecular bridge between memory acquisition and consolidation in a spatio-temporally specific manner upon dopaminergic modulation. However, this hypothesis has not been directly tested in behaving animals.

To investigate the mechanisms of long-term memory consolidation in *Drosophila*, we employed a courtship memory consolidation paradigm. Courtship learning can induce either short- or long-lived courtship memories, depending on the duration of the learning experience (McBride et al., 1999; Siegel and Hall, 1979). For example, exposing a *Drosophila* male to an unreceptive mated female for 5–7 hr leads to a long-term suppression of his courtship preferentially toward a mated, but not a virgin, female for at least 24 hr. We were able to prevent the expression of this long-term memory by blocking the aSP13 dopaminergic neurons several hours after their initial involvement during learning experience with a mated female (Keleman et al., 2012). Further, exposure to a mated female for only 1 hr results in short-term memory, but not long-term memory; however, by stimulating the same class of dopaminergic neurons, aSP13, many hours after this exposure, we were able to transform short-term memory into long-term memory. Having revealed that long-term memory consolidation requires late activation of the same class of dopaminergic neuron, aSP13, that hours earlier is necessary for memory acquisition, we explored it further using genetic, molecular, and behavioral analyses. We established that the DopR1 type of receptor, shown previously to be necessary for memory acquisition, is also required for late long-term memory consolidation in the mushroom body (MB) γ neurons. We determined that Orb2A is localized at synapses in the MB neurons and functions during memory acquisition to mark potentially specific MB neurons and specific synapses for eventual long-term memory consolidation. Upon subsequent late dopaminergic pathway activation, Orb2A recruits Orb2B into complex to regulate translation of CaMKII, a key protein involved in triggering memory persistence (Ashraf et al., 2006; Coultrap and Bayer, 2012).

RESULTS

Dopaminergic Stimulation after Learning Is Sufficient to Consolidate Short-Term Memory into Long-Term Memory

We employed a paradigm for courtship memory consolidation to investigate the molecular basis and spatiotemporal relationships between the two key processes of long-term memory formation, memory acquisition and its consolidation, in the *Drosophila* male. We combined a learning experience sufficient to establish courtship short-term memory, but not long-term memory, with a subsequent stimulation of the dopaminergic pathways, which is thought to induce local protein synthesis at synapses (Smith et al., 2005). We starved naive males for 16 hr, trained them for 1 hr with mated female, and then activated dopamine pathways globally by feeding the animals with dopamine for 23 hr (Riemensperger et al., 2011). This resulted in robust long-term memory, in the form of a strong suppression of their courtship toward mated females in comparison to virgin females when tested 24 hr later. This memory was quantified as a learning index (LI), which measures the extent of the courtship suppression (Figure 1; Table S1). By contrast, training for 1 hr alone induced normal short-term memory, but not long-term memory, and dopaminergic activation alone did not induce long-term memory. Thus post-acquisition dopamine stimulation consolidates short-term memory into long-term memory.

Subset of PAM-DA Neurons, aSP13, Mediates Late Long-Term Memory Consolidation in a Protein-Synthesis-Dependent Manner

DA neurons are organized in the fly brain into 15 clusters, with the PPL1-DA and PAM-DA cluster innervating the MB lobes, a neuropil consisting of the axonal projections of the intrinsic MB cells called Kenyon cells (KC) (Mao and Davis, 2009). A subset of the

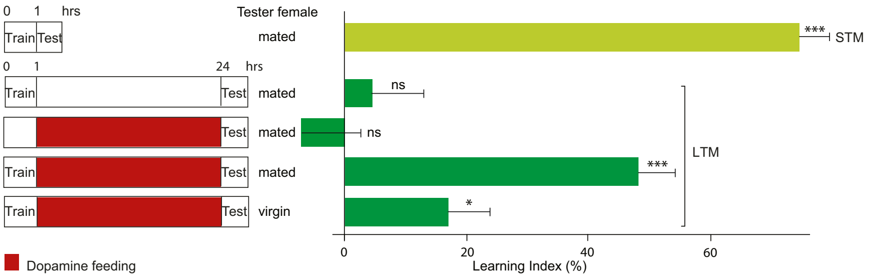

Figure 1. Post-learning Global Activation of Dopamine Pathways Is Sufficient to Consolidate Short-Term Memory into Long-Term Memory

The plots indicate mean learning indices ± SEM of the wild-type *Canton-S* males tested in single-pair assays with mated or virgin females, either 24 hr after being starved for 16 hr, trained with mated female for 1 hr, and fed with dopamine for 23 hr (if indicated; LTM; dark green bars) or immediately after training (STM; light green bar). p values are for H_0 LI = 0; *p < 0.05; ***p < 0.001. See Table S1.

PAM-DA neurons was previously implicated in courtship memory encoding (Keleman et al., 2012). Given that global post-acquisition stimulation of dopamine pathways was sufficient to consolidate short-term courtship memory into a long-lasting one, we asked whether delayed activation of PAM-DA neurons might be sufficient for consolidation of courtship short-term memory into long-term memory. To test whether and when activity of the PAM-DA neurons is sufficient for long-term memory consolidation, we thermogenetically manipulated their activity by a temperature-gated cation channel, *TrpA1* (Rosenzweig et al., 2005). We expressed *UAS-TrpA1* with *HL09-Gal4* (Claridge-Chang et al., 2009) in a large population of DA neurons, including PAM-DA cluster and few neurons of PPL1-DA cluster, in combination with training for short-term memory.

Males expressing TrpA1 were incubated at 32°C for 2 hr at various time points after 1-hr training with a mated female. Flies, which were incubated at 32°C 8–10 hr after training fully consolidated short-term memory into long-term memory, in contrast to appropriate genetic control flies or flies that were switched to 32°C at other time points or control flies that remained at 22°C throughout (Figures 2Ai and 2Aii; Table S2). Importantly, this consolidated form of memory was dependent on de novo protein synthesis, because feeding the males with the protein synthesis inhibitor cycloheximide prevented the PAM-DA-stimulation-induced long-lasting memory (Figure 2Aiii; Table S2). Given our previous results showing that PAM-DA neurons are necessary for short-term memory acquisition, these results suggest that delayed activation of the same PAM-DA neurons between 8 and 10 hr after training is sufficient to consolidate short-term memory into a protein-synthesis-dependent long-term memory.

The PAM-DA cluster consists of over 100 neurons, a subset of which expresses *fruitless* (*fru*), a gene causally linked with multiple aspects of male courtship behavior (Dickson, 2008). The specific fru^+ class of PAM-DA neurons innervating MB γ neuropil, aSP13, was implicated recently in courtship memory encoding (Keleman et al., 2012). To investigate whether activity of the fru^+ set of PAM-DA neurons is sufficient for memory consolidation after learning, we restricted expression of *UAS-TrpA1* to two fru^+ neurons using *TH-Gal4*, *UAS > stop > TrpA1*, and fru^{FLP} (where ">" represents an FRT site, the target of the Flp recombinase; Yu et al., 2010). Males expressing *TrpA1* in fru^+ *TH-Gal4* neurons displayed suppressed courtship toward mated females at 24 hr post-training when incubated at 32°C between 8 and 10 hr after learning, indicating that they robustly formed long-term memory (Figure 2B; Table S3). When we restricted expression of *TrpA1* exclusively to a single class of aSP13 neurons, using *VT5526-LexA*, *LexAop-TrpA1* (Figure S1; B.J. Dickson, personal communication) males incubated at 32°C from 8 to 10 hr post-training fully consolidated long-term memory in contrast to genetic control under the same conditions or control flies that remained at 22°C throughout (Figure 2B; Table S3). These results suggest that post-training activation of the dopaminergic neurons, aSP13, is sufficient for consolidating short-term memory into long-term memory.

To test whether late post-acquisition activity of the aSP13-DA neurons is also necessary for formation of courtship long-term memory, we performed a complementary set of experiments. We trained males for 7 hr with recently mated females (sufficient to result in long-term memory) and silenced the same aSP13 neurons with a temperature-sensitive shibire mutant, *LexAop-*shi^{ts}, between 8 and 11 hr after onset of training. Males expressing shi^{ts} under the control of *VT5526-LexA* and incubated at 32°C continued to court mated females vigorously, thus failing to display long-term memory at the 24-hr test point. In contrast, genetic control animals incubated at 32°C and males that remained at 22°C displayed normal long-term memory (Figure 2C; Table S4). These results indicate that activity of the aSP13-DA neurons is required between 8 and 11 hr after initial learning for long-term memory consolidation. Thus, delayed activation of a specific neural subset is both necessary and sufficient for long-term memory consolidation of courtship learning in *Drosophila*.

DopR1 Is Necessary for Long-Term Memory Consolidation

In rodents, dopamine-mediated memory persistence requires the adenylyl cyclase stimulatory D1-like type of dopamine receptors (Rossato et al., 2009). There are four dopamine receptors in *Drosophila*: two D1-like dopamine receptors, DopR1 and DopR2 (Hearn et al., 2002; Kim et al., 2003); one D2-like type receptor, DD2R (Draper et al., 2007); and recently identified DopEcR (Srivastava et al., 2005). To determine which dopamine receptor has a role in long-term courtship memory consolidation, we focused our analysis on DopR1 and DopR2 because DD2R is not expressed in the MB and DopEcR does not act as a dopamine receptor in courtship-suppression learning (Ishimoto et al., 2009). We tested null mutants for either type of receptor, $DopR1^{attp}$ or $DopR2^{attp}$, in long-term memory and memory-consolidation paradigms (Keleman et al., 2012).

Males lacking *DopR1* were unable to form long-term courtship memory after 7 hr of training. In contrast, *DopR2* mutants displayed normal long-term memory (Figure 3A; Table S5). To confirm that long-term memory deficit in the *DopR1* mutants is

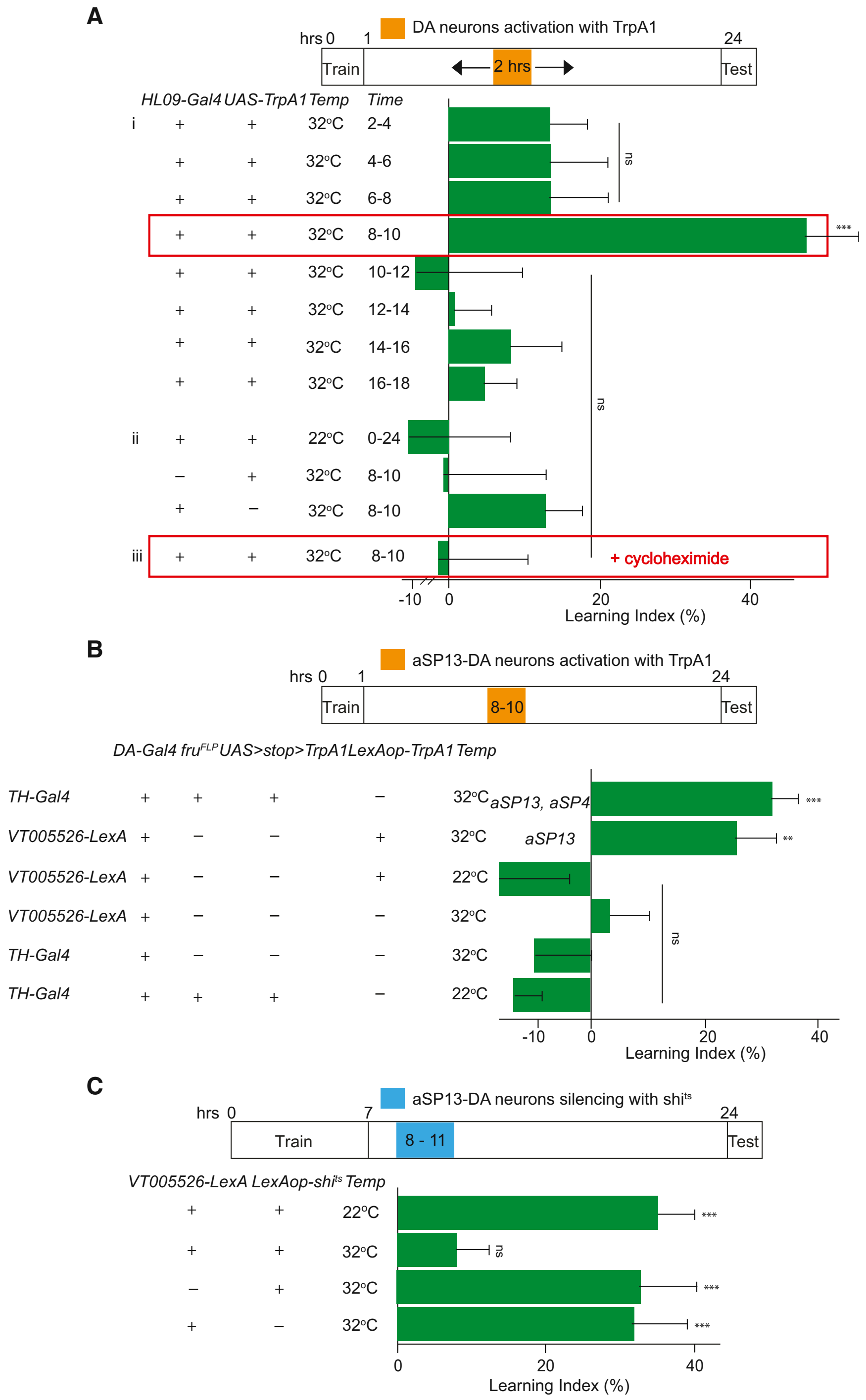

Figure 2. Subset of the PAM-DA Neurons, aSP13, Consolidates Short-Term Memory into Long-Term Memory in a Protein-Synthesis-Dependent Manner

(A) Post-acquisition activation of the DA neurons mediates protein-synthesis-dependent memory consolidation. Males of the indicated genotypes were tested in single-pair assays with mated females 24 hr after being trained for 1 hr with a mated female and warmed at 32°C for 2 hr at the indicated time points (i); experimental control males, which stayed at 22°C all the time; and the genetic control animals, which were warmed at 32°C for 2 hr between 8 and 10 hr after learning (ii); and males fed with the cycloheximide during activation with TrpA1 between 8 and 10 hr after 1 hr training (iii). p values are for H_0 LI = 0; ***$p < 0.001$. See Table S2.

(B) Subset of the aSP13-DA neurons is sufficient for the long-term memory consolidation. Males of the indicated genotypes were tested in single-pair assays with mated females 24 hr after training for 1 hr with a mated female and being warmed at 32°C (except control males which stayed at 22°C) between 8 and 10 hr after training. p values are for H_0 LI = 0; **$p < 0.01$; ***$p < 0.001$. See Table S3.

(C) Post-acquisition silencing of the aSP13-DA neurons prevents long-term memory formation. Males of the indicated genotypes were tested in single-pair assays with mated females 24 hr after training for 7 hr with a mated female and being warmed at 32°C (except control males, which stayed at 22°C) between 8 and 11 hr after training. p values are for H_0 LI = 0; ***$p < 0.001$. See Table S4.

The plots indicate mean learning indices ± SEM.

indeed due to loss of *DopR1* function, we analyzed *DopR1*^{res} flies where the deleted genomic region was reintegrated by site-specific transgenesis (Keleman et al., 2012). These flies performed just as well as wild-type animals, suggesting that DopR1, but not DopR2, is required for long-term memory (Figure 3A; Table S5). However, given that DopR1 receptor is required for acquisition of the courtship short-term memory (Keleman et al., 2012), the impairment of long-term memory in *DopR1* mutants might be due to its involvement in this early phase of memory formation: thus, these results alone do not prove DopR1's role in memory consolidation. To address explicitly the requirement of DopR1 in long-term memory after memory acquisition, we fed wild-type flies and *DopR2*^{attp} mutants (lacking DopR2, but not DopR1) after 7-hr training for long-term memory with the antagonist (SCH23390) specific for both receptors (Gotzes et al., 1994). These flies did not form long-term memory in contrast to animals that were not fed with the antagonist, suggesting that DopR1 has a post-acquisition role in long-term memory (Figure 3A; Table S5).

To test both dopamine receptors in memory consolidation paradigm, we tested *DopR1* and *DopR2* mutants for courtship suppression after being trained for short-term memory in combination with dopamine feeding. Mutants for *DopR1* were unable to consolidate long-term memory, whereas the *DopR2* mutants performed equally well as the wild-type animals (Figure 3B; Table S6). To dissociate the role of DopR1 in memory acquisition and memory consolidation, we fed wild-type males and

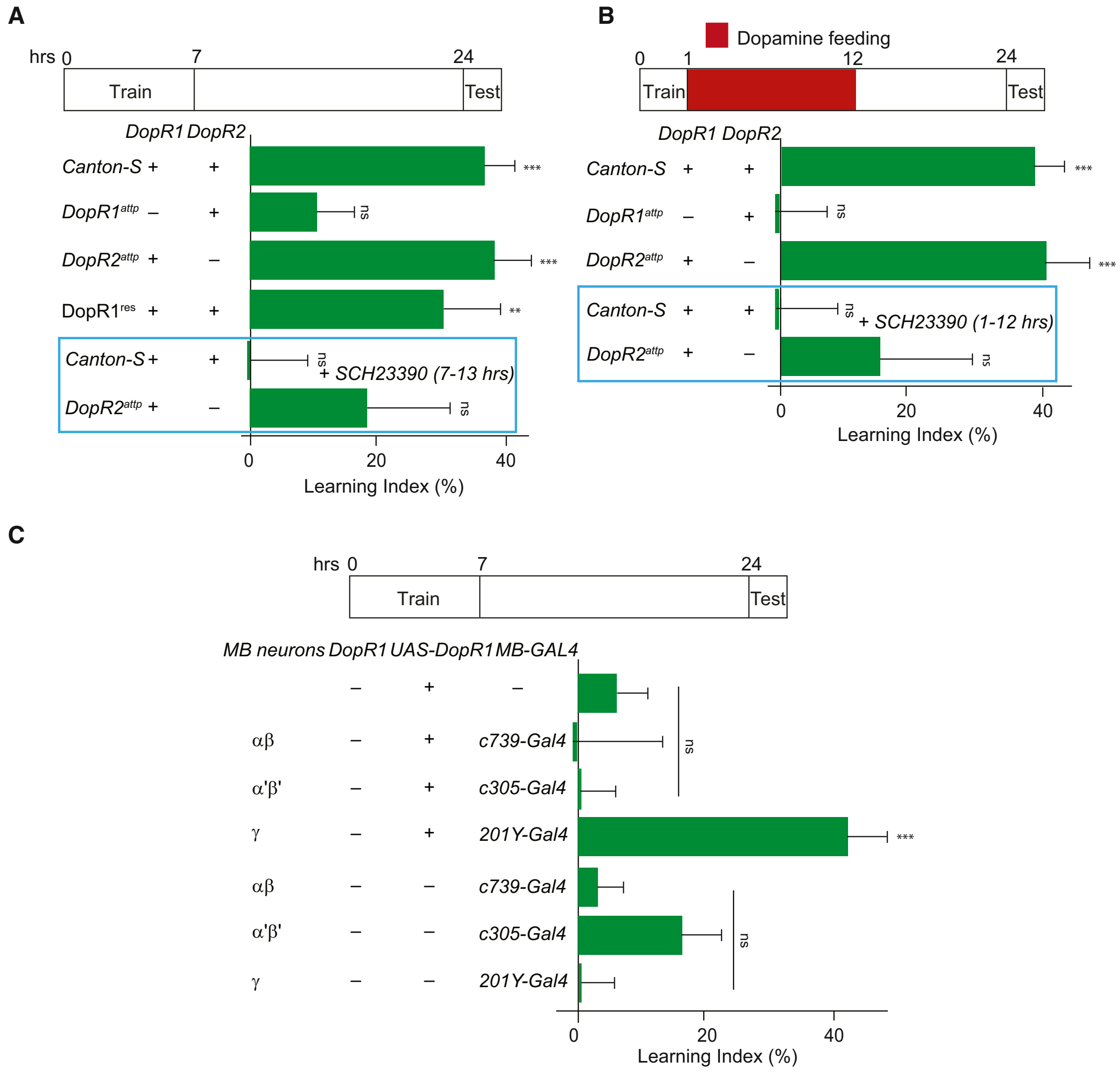

Figure 3. DopR1 Is Necessary in the MB γ Neurons for Long-Term Memory Consolidation

(A) Post-acquisition inactivation of DopR1 impairs long-term memory. Males of the indicated genotypes were tested in single-pair assays with mated females 24 hr after being trained for 7 hr with a mated female and fed with SCH23390 for 6 hr if indicated. p values are for H_0 LI = 0; ***$p < 0.001$; **$p < 0.01$. See Table S5.

(B) DopR1 is required for dopamine-mediated memory consolidation. Males of the indicated genotypes were tested in single-pair assays with mated females 24 hr after being starved for 16 hr, trained for 1 hr with a mated female, and fed with dopamine and SCH23390 as indicated between training and test. p values are for H_0 LI = 0; ***$p < 0.001$. See Table S6.

(C) DopR1 functions in long-term memory in the MB γ neurons. Males of the indicated genotypes were tested in single-pair assays with mated females 24 hr after being trained for 7 hr with a mated female. p values are for H_0 LI = 0; ***$p < 0.001$. See Table S7.

The plots indicate mean learning indices ± SEM.

DopR2^{attp} mutants with the SCH23390 in addition to dopamine. These males, in contrast to animals fed only with dopamine, did not suppress their courtship toward mated females during test, thus failing to display long-term courtship memory (Figure 3B; Table S6). These results indicate that DopR1 has a role in the consolidation of short-term memory into long-term memory upon post-acquisition dopamine stimulation.

DopR1 is required in the MB γ neurons for short-term courtship memory encoding (Keleman et al., 2012). To investigate in which MB neurons DopR1 is required for memory persistence, we expressed *UAS-DopR1* transgene in the *DopR1* mutant background using MB lobe-specific Gal4s (Keleman et al., 2012) and tested them for long-term memory. Memory was fully rescued when DopR1 was provided back in the γ, but not α, β and α', β' MB neurons, indicating MB γ neurons as a likely site of long-term memory consolidation (Figure 3C; Table S7).

Orb2 Mediates Long-Term Memory Consolidation Downstream of DopR1

Feeding animals with dopamine activates dopaminergic pathways globally, which leads to formation of Orb2 protein complexes consisting of Orb2A and Orb2B. The Orb2 complex

OPEN
ACCESS
CellPress

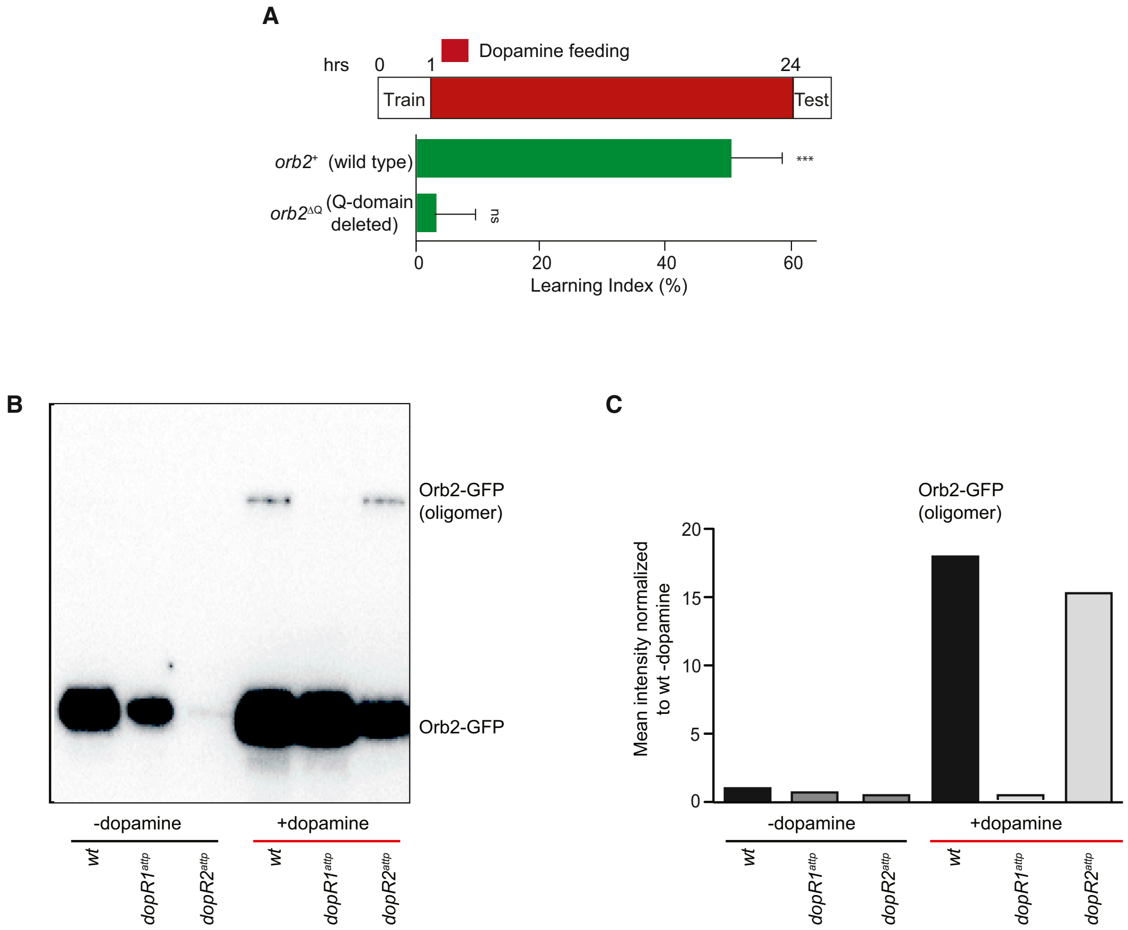

Figure 4. Orb2 Mediates Long-Term Memory Consolidation Downstream of DopR1

(A) Orb2 is required for dopamine-mediated memory consolidation. The plots indicate mean learning indices ± SEM of males of the indicated genotypes tested in single-pair assays with mated females 24 hr after being starved for 16 hr, trained with a mated female for 1 hr, and fed with dopamine. p values are for H_0 LI = 0; ***p < 0.001. See Table S8.

(B) DopR1 mediates Orb2 oligomer formation. Head extracts from adult flies of the indicated genotypes were analyzed by IP and WB for presence of the $Orb2^{GFP}$ complexes, after being starved for 16 hr and fed with dopamine as indicated.

(C) WB signal (from C), corresponding to the Orb2-GFP oligomers, has been quantified using Fiji-ImageJ. The values on y axis represent the mean intensity normalized to the wild-type not treated with dopamine (wt − dopamine). See Table S9.

correlates strictly with the ability of males to form courtship long-term memory (Krüttner et al., 2012; Majumdar et al., 2012). To test whether Orb2 complexes are required for dopamine-mediated memory consolidation, we used an endogenously modified *orb2* mutant allele. This mutant lacks the Q domain ($orb2^{\Delta Q}$) and has been previously shown to be dispensable for short-term memory but critical for both Orb2 complex formation and the maintenance of the courtship memory after 6 hr (Keleman et al., 2007; Krüttner et al., 2012). $orb2^{\Delta Q}$ mutants were unable to consolidate short-term memory into a memory lasting 24 hr upon feeding with dopamine, in comparison to the animals bearing the wild-type allele ($orb2^{+}$; Figure 4A; Table S8).

To investigate which type of dopamine receptor is mediating the Orb2 complex formation and hence long-term memory consolidation, we examined whether the propensity of Orb2 to form complexes depends on either DopR1 or DopR2 receptor. We investigated the endogenous Orb2 protein tagged with the GFP tag ($Orb2^{GFP}$) in immunoprecipitates from brains of the *DopR1* or *DopR2* mutant flies upon stimulation with dopamine. As predicted, $Orb2^{GFP}$ complex was not detected in brain extracts from the animals that were not fed with dopamine (both the wild-type and mutants). In contrast, $Orb2^{GFP}$ complex was detected in brain extracts from the wild-type animals fed with dopamine, but not in *DopR1* mutants. Although levels of the Orb2 protein were lower in both mutants in comparison to the wild-type animals, particularly in the animals lacking *DopR2*, the propensity to form Orb2 complexes seems not to be affected (Figures 4B and 4C; Table S9). These results suggest that DopR1 functions upstream of Orb2 complex formation and hence memory consolidation.

Orb2A Is Localized Mainly to Synapses in MB Neurons

Light microscopy studies using antibodies against the GFP tag fused to the endogenous Orb2 protein determined that Orb2A

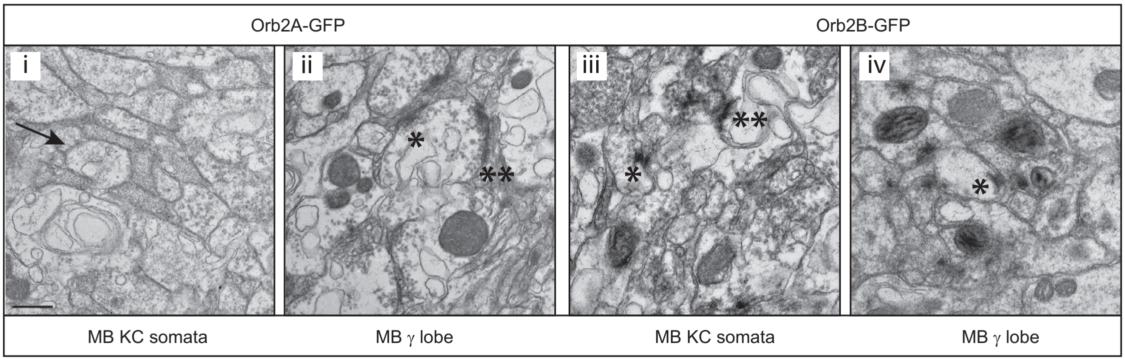

Figure 5. Orb2A Is Localized in Synapses of the MB γ Lobe

Immuno-EM of the *orb2*^{Orb2AGFP} and *orb2*^{Orb2BGFP} heterozygous brains. The sagittal sections of the brain in the region of the KC somata and the tip of the MB γ lobe were analyzed. (i) Orb2A^{GFP} is absent from the neuronal cell bodies (arrow) of the Kenyon cells. (ii) Orb2A^{GFP} labeled by DAB precipitates is present in T-bars (asterisk) and active zones (double asterisk) in the MB γ lobe synapses. (iii) Orb2B^{GFP} labeled by DAB precipitates is detected in the KC cell bodies including T-bars (asterisk) and active zones (double asterisk). (iv) Orb2B^{GFP} labeled by DAB precipitates is present in the MB γ lobe including T-bars (asterisk). In all panels, scale bars represent 500 nm.

and Orb2B isoforms are localized in the nervous system in distinct patterns. Orb2B appears to be widely distributed throughout various regions of the nervous system, including the lobes, calyces, and soma of the MB. In neurons, Orb2B is expressed very broadly, including in ribonucleoprotein transport granules (RNPs) (Krüttner et al., 2012; Majumdar et al., 2012). In contrast, endogenous Orb2A was expressed at levels undetectable by confocal microscopy. When expressed with GFP-tagged genomic transgene rescue, Orb2A was detected at very low levels (Majumdar et al., 2012). Consistent with the genetic data that revealed a functional requirement for Orb2A in long-term memory, we hypothesized that Orb2A is expressed in the adult brain at very low levels or/and in very few cells and only in a specific cellular compartment, at the synapses, and therefore undetectable by light microscopy.

Using immuno-electron microscopy against the GFP tag on the endogenous Orb2A and Orb2B proteins encoded by *orb*^{Orb2AGFP} and *orb*^{Orb2BGFP}, respectively, we determined their subcellular localization (Krüttner et al., 2012). We examined KC somata and the output region of the MB, tip of the γ lobe, innervated by the aSP13 neurons in the brains of viable heterozygous *orb*^{Orb2AGFP} and *orb*^{Orb2BGFP} flies. We detected Orb2A protein present in a pattern distinct to that of the Orb2B protein. Orb2B is broadly expressed in the KC cell bodies and axons of the γ neurons, including synapses, whereas Orb2A is excluded from the neuronal cell bodies and is almost exclusively present in synapses of the MB γ lobe (Figure 5). These results imply that Orb2 isoforms, previously shown to function in long-term memory through distinct mechanisms (Krüttner et al., 2012), likely play distinct roles in long-term memory.

Orb2A Is Required during Memory Acquisition and Orb2B during Memory Consolidation

To investigate the temporal requirement of Orb2 isoforms in long-term memory, we manipulated in a temporal manner expression of either Orb2 isoform using TARGET expression system. TARGET uses ubiquitous expression of *Gal80*^{ts} to conditionally suppress a Gal4-driven transgene; at 18°C, Gal80 inhibits Gal4 activity and expression of the transgene, but at 27°C, *Gal80*^{ts} is inactive and the transgene is expressed (McGuire et al., 2004). Because it is thought that Orb2 functions in the MB γ neurons for long-term memory (Keleman et al., 2007), we used TARGET in combination with *MB247-Gal4*, which drives expression in the MB γ and αβ neurons, as this genetic combination resulted in the healthy progeny able to perform courtship-learning assays.

To examine temporal requirement of Orb2A and its Q domain, we expressed in the MB neurons either wild-type Orb2A (*UAS-orb2AGFP*) or Orb2A with the Q domain deleted (*UAS-orb2ΔQGFP*) under control of *MB247-Gal4* and temperature-gated *tub-Gal80*^{ts} in the flies lacking the A isoform and the Q domain in Orb2B (*orb2B*$^{\Delta Q}$) and hence unable to form Orb2 complexes and long-term memory. We performed the IP and WB against the GFP tag to analyze the on/off kinetics of Orb2 protein expression using TARGET system. Orb2 protein is expressed at high levels within 3 or 4 hr after temperature shift to 27°C and is not detectable at the time of memory retrieval (Figure S2). Flies had fully rescued long-term memory both when kept at 27°C throughout adulthood and when shifted to 27°C for the duration of the training (Figure 6; Table S10) and only when a wild-type, but not the Q-domain-deleted, Orb2A was present. These results suggested that the Orb2A isoform and its Q domain are required in the MB neurons during memory acquisition for long-term memory formation.

Because of the role Orb2B plays during development, animals lacking this isoform do not survive to adulthood; thus, we could not directly assess its temporal requirement in long-term memory. Therefore, we employed mutant flies in which the RNA-binding domain of Orb2B was substituted with the RNA-binding domain of the mouse homolog mCPEB2 (*orb2*^{mCPEB2RBD}; Krüttner et al., 2012). These viable (but unable to form long-term memory) mutant flies allowed us to assess the temporal requirement of Orb2B and its RNA-binding domain in memory independently of its role during development. Flies expressing wild-type Orb2B (*UAS-orb2BRBD*) had normal long-term memory when

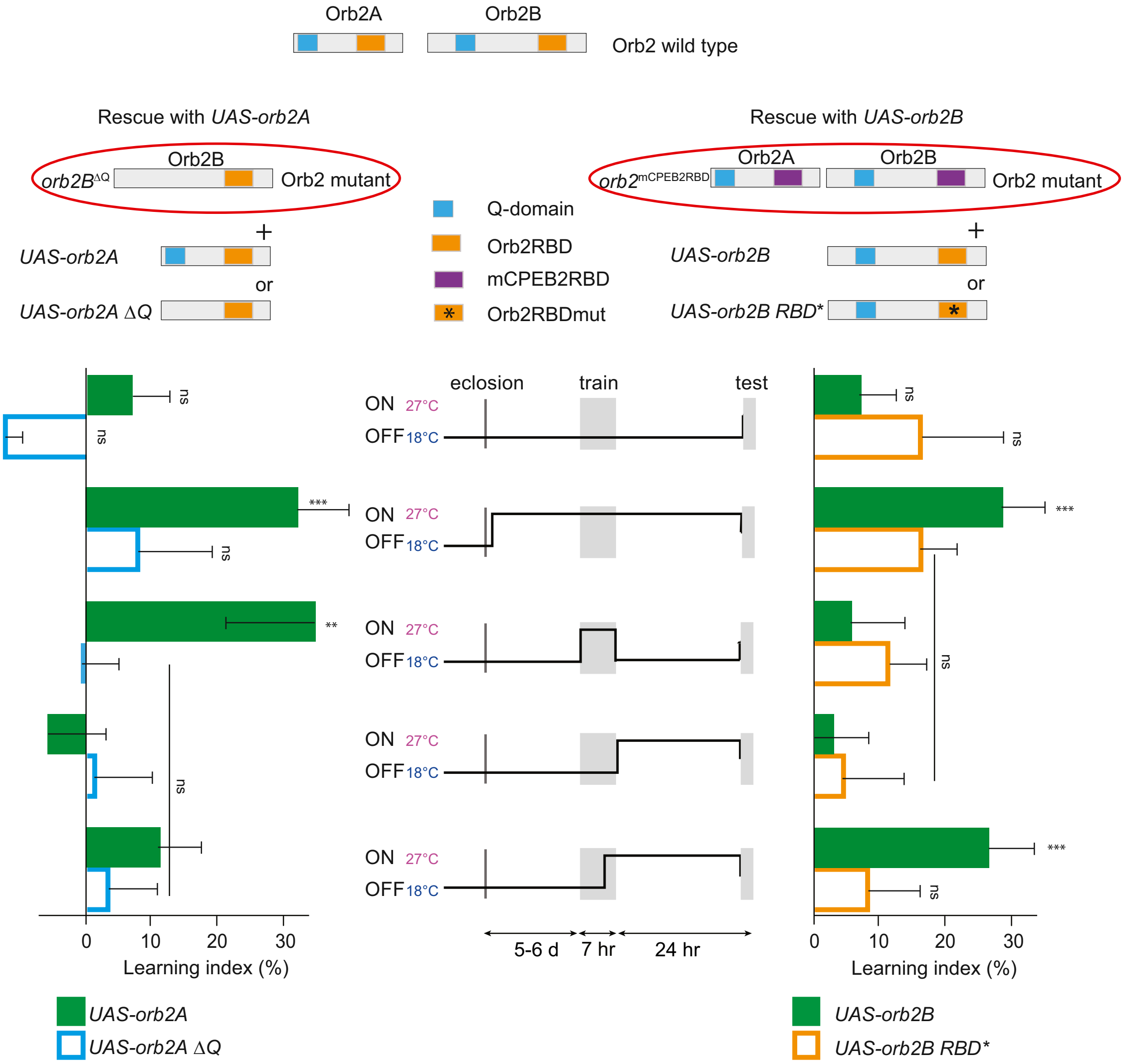

Figure 6. Orb2A Is Required during Memory Acquisition and Orb2B during Memory Consolidation

(Upper panel) Schematic of the rescue experiment with depicted domain organization of the proteins involved. (Lower panel) The plots indicate mean learning indices ± SEM of the temporal rescue of the *orb2*$^{Orb2B\Delta Q}$ and *orb2*^{mCPEB2RBD} mutants with either *UAS-orb2A/UAS-orb2A*ΔQ or *UAS-orb2B/UAS-orb2BRBD** under control of *MB247-Gal4* and *tubGal80ts*, trained and tested in single-pair assays with mated females for long-term memory and treated according to the regime outlined in the center of the panel. p values are for H_0 LI = 0; ***p < 0.001; **p < 0.01. See Table S10.

kept at 27°C throughout adulthood and when shifted to 27°C ~2 hr before the end of training. They did not form long-term memory when kept at 27°C during training only and when switched to 27°C right after training. Importantly, this memory was dependent on the RNA-binding domain because the males with the RNA-binding domain mutated (*UAS-orb2BRBD**) could not form long-term memory in any condition (Figure 6; Table S10). Therefore, we conclude that presence of Orb2B is dispensable during the training and shortly after but is necessary continuously about 2 hr after training. Together, these results suggest that the Orb2A isoform, which is localized to MB synapses, is necessary during memory acquisition, whereas the Orb2B isoform (recruited into complexes with Orb2A) is necessary during long-term memory consolidation.

Orb2 Regulates Translation of CaMKII in MB Neurons

Dopamine regulates the expression of proteins essential for long-lasting memories (Berke et al., 1998), such as calcium/calmodulin-dependent kinase (CaMKII). CaMKII translation at synapses is dependent on neuronal activity both in mouse and *Drosophila* (Ashraf et al., 2006; Coultrap and Bayer, 2012) and is conferred by its 3′ UTR, which is recognized by CPEB proteins in mouse (Wu et al., 1998). *Drosophila* CaMKII has been identified as an Orb2 mRNA target (our unpublished results), and Orb2 regulates its translation by binding to the specific sequence in the 3′ UTR (Figure 7A; Table S11).

Given that CaMKII is a key molecule implicated in memory persistence and Orb2 regulates translation of CaMKII, we

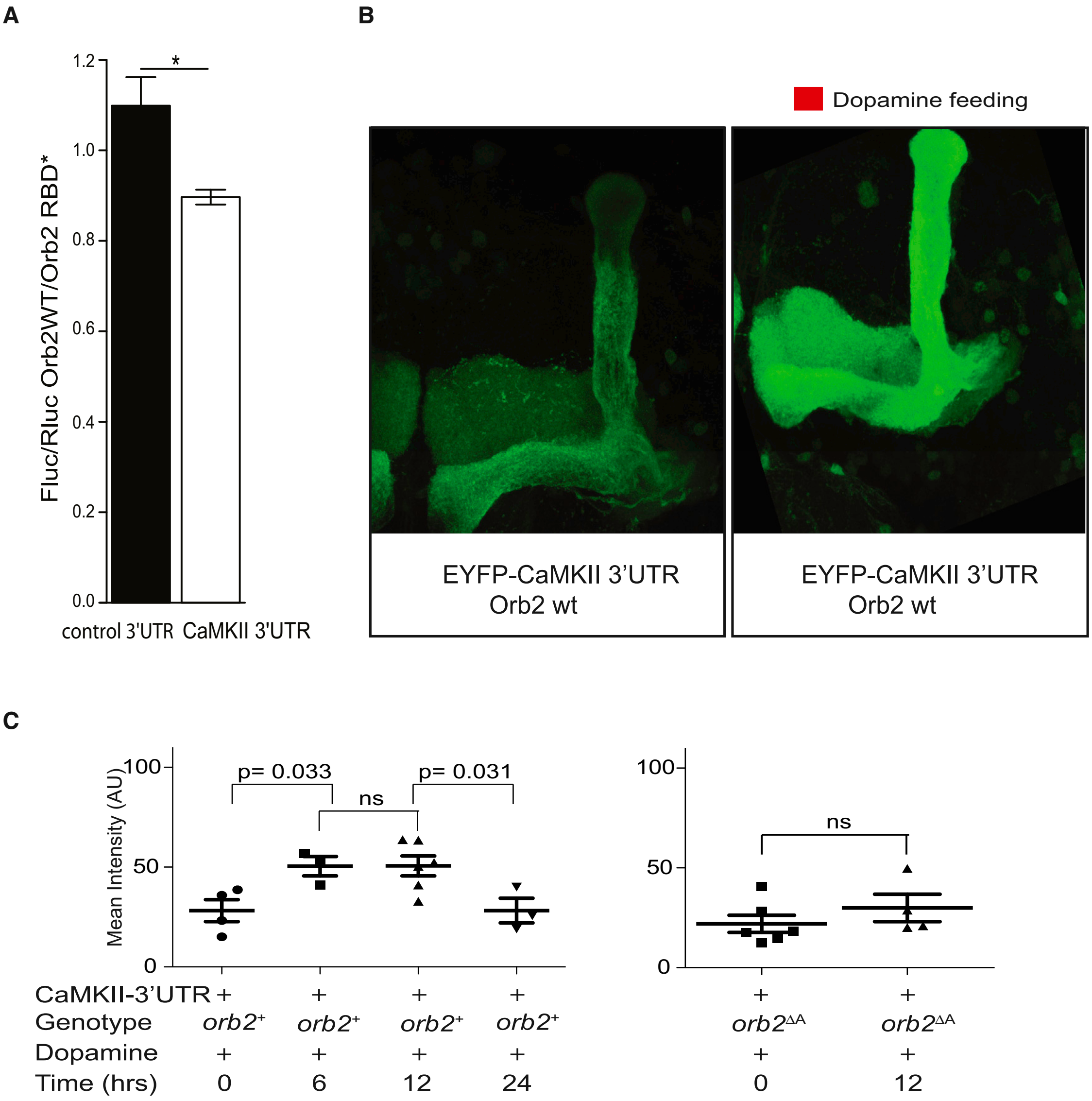

Figure 7. Orb2 Regulates Synthesis of CaMKII in MB Neurons

(A) Orb2 regulates translation of CaMKII through its 3′ UTR. Translation of the Firefly luciferase (Fluc) reporter tethered to the CaMKII 3′ UTR is suppressed by Orb2, but not when tethered to the control 3′ UTR, which does not contain Orb2-specific binding sequence. The values on y axis represent the ratio of the normalized Fluc to Rluc fluorescence in the presence of wt Orb2 to the Fluc/Rluc fluorescence in the presence of Orb2RBD* with RBD mutated. *$p < 0.05$. See Table S11.

(B) Representative confocal projections of the MB (lobes) expressing *UAS-EYFP-CaMKII-3′UTR* with the *MB247-Gal4* and stained with the anti-GFP antibodies. The adult brains were either unstimulated (left panel) or stimulated (right panel) by feeding with dopamine.

(C) *UAS-EYFP-CaMKII-3′UTR* was expressed in the MB neurons with the *MB247-Gal4*. Intensity of fluorescence was measured in the MB γ lobe of the adult brains either wild-type (*orb2*$^{+}$; left panel) or orb2 mutant (*orb2*$^{\Delta A}$; right panel) stimulated by feeding with dopamine at the indicated time intervals. p values are for H_0 ftx = ft0. See Table S12.

investigated whether Orb2 might function in memory consolidation by regulating synthesis of CaMKII. We expressed a fluorescent reporter of CaMKII translation, CaMKII 3′ UTR appended to the EYFP coding region (*UAS-EYFP*$^{3'UTR}$), in the α, β, γ MB neurons using *MB247-Gal4* (Ashraf et al., 2006). We monitored the change in intensity of the EYFP signal in the MB γ neurons upon neuronal stimulation with dopamine in comparison to unstimulated control brains.

We observed a striking increase of the EYFP signal after stimulation with dopamine. The EYFP signal was highest at 6 and 12 hr post-dopamine stimulation in comparison to unstimulated control brains at baseline. Importantly, we did not observe a dopamine-induced EYFP increase in an Orb2 mutant lacking the Orb2A isoform (*orb2*$^{\Delta A}$; Figures 7B and 7C; Table S12), whose Q domain is required for Orb2 complex formation (Krüttner et al., 2012). Thus, these results suggest that Orb2

complexes induced upon dopamine stimulation regulate translation of CaMKII and possibly other molecules essential for synaptic plasticity.

DISCUSSION

Our results demonstrate that the process of long-term memory consolidation in *Drosophila* requires activation of the same neural pathway that, hours earlier, is required for memory acquisition. Specifically, we identified a subset of PAM-DA neurons (aSP13) whose activation mediates both memory acquisition and late memory consolidation. This permitted us to examine how memory is maintained during the interval between memory acquisition and memory consolidation. First, we established that aSP13 neurons mediate both memory acquisition and memory consolidation through the activation of the DopR1 type of receptor and through *Drosophila* CPEB, Orb2, in the MB γ neurons. Then, we determined that the Orb2A isoform is localized mainly to synapses in the MB neurons and is required during memory acquisition, tagging the relevant neurons and potentially their synapses for subsequent memory consolidation, whereas Orb2B, recruited into complexes with Orb2A, is required during memory consolidation. Finally, we show that, together, they regulate the activity-dependent synthesis of CaMKII, a key protein involved in the molecular basis of memory persistence (Coultrap and Bayer, 2012; Lucchesi et al., 2011; Redondo et al., 2010).

Delayed post-learning reactivation of neural pathways has been shown to exist in vertebrates (Buzsáki, 1998; Foster and Wilson, 2006; Wilson and McNaughton, 1994). Spontaneous neuronal replay after learning occurs both in the awake and sleeping states (Carr et al., 2011; Wilson and McNaughton, 1994), but the causal link between replay and memory consolidation has not been firmly established. Interfering with sharp wave ripples (SWRs), which are temporally correlated with neuronal replay in awake animals (Buzsáki et al., 1992), impaired spatial memory formation in rats (Jadhav et al., 2012). These results suggested that replay might mediate memory consolidation; however, they could not rule out that other effects of SWRs may be critical for memory consolidation. Our results that courtship memory acquisition and consolidation in *Drosophila* are mediated by activation of the same neuronal pathway provides further evidence that reactivation might play a key role in memory consolidation. An emerging view is that multiple types of neural signals are involved in memory formation, including neural representations of the specific content to be stored, along with signals pertaining to the importance or valence of an event, which may influence whether the content becomes consolidated. The aSP13 pathway implicated in the present work may be of the latter type, given that it is a neuromodulatory pathway. Prior work in rodents has implicated both types of signals in memory formation: for example, SWRs are thought to carry the content of a spatial trajectory (Carr et al., 2011) whereas the neuromodulatory pathways are thought to convey the salience of the content (Musso et al., 2015; Waddell, 2010).

There are emerging clues regarding the molecular bases underlying delayed neuronal-reactivation-dependent memory consolidation. Recently, a requirement of NMDA receptor reactivation for memory consolidation has been explored in rodents (Wang et al., 2006). This led to the formulation of the synaptic re-entry reinforcement hypothesis (SRR), which posits that memory consolidation requires delayed reactivation of the NMDA receptor to convert short-term memory into long-term memory. Interestingly, one of the signaling molecules downstream of NMDA receptor is CaMKII, which is believed to be responsible for potentiating the synapses involved in learning (Nicoll and Malenka, 1999). Our results are consistent with the SRR but involving DopR1 instead of NMDAR, as we found that the *Drosophila* DopR1, which is required during memory acquisition (Keleman et al., 2012), seems to be also necessary for late memory consolidation. This is consistent with a recent finding that DopR1 expression in the MB γ neurons is sufficient to fully support both short- and consolidated long-term memory in *Drosophila* (Qin et al., 2012). If the SRR hypothesis is correct (i.e., synaptic re-entry leads to reinforcement), what molecular change signifies that an "initial entry" has previously occurred in a synapse, such that another synaptic event counts as "re-entry"?

The synaptic tagging and capture hypothesis has provided a conceptual framework for how relevant information might be stored in the intervening time period between memory acquisition and memory consolidation and how specific synapses eventually come to store a given memory. The ability to temporally dissociate memory acquisition and its consolidation in the courtship memory consolidation paradigm allowed us to investigate the molecular basis of this hypothesis in learning *Drosophila*. Our results that the synaptically localized Orb2A isoform and its Q domain are required during memory acquisition in MB neurons for subsequent long-term memory consolidation support the likelihood that synaptic Orb2A might act to tag the specific synapses for later memory consolidation. At present, we cannot distinguish between the possibilities that this tagging is an effect of a synapse-specific post-translational modification of Orb2A or its mere presence at a synapse (White-Grindley et al., 2014). Thus, during memory acquisition, Orb2A or a modification thereof might mark activated synapses as potential sites for subsequent memory storage. Only in those synapses where the delayed activation occurred would Orb2A recruit Orb2B (and possibly its associated mRNAs; Krüttner et al., 2012) into translationally active protein complexes (Si et al., 2003b) to regulate synthesis of proteins essential for the long-term memory, such as CaMKII.

In this work, we provide evidence that the late activation of the same neuronal and molecular pathways that are necessary and sufficient for early memory acquisition is also necessary and sufficient for late memory consolidation in *Drosophila*. These findings confirm principles that were strongly implied by work in mammals (Carr et al., 2011; Wang et al., 2006; Wilson and McNaughton, 1994) and extend this paradigm to invertebrates. Taking advantage of the tools available for the molecular and circuit analysis in *Drosophila*, we provide a functional link between occurrence of the delayed neural pathway activation and memory consolidation and start to identify the molecular and circuit mechanisms underlying this consolidation. The occurrence of these phenomena in evolutionarily distinct species implies that

delayed activation might serve a key algorithmic role in adaptive learning. Moreover, a high degree of conservation of the involved molecules (Theis et al., 2003) suggests that the molecular mechanisms uncovered in flies might be broadly utilized in the animal kingdom.

EXPERIMENTAL PROCEDURES

Fly Stocks

Flies were maintained on conventional cornmeal-agar medium under a 12 hr light/dark cycle at 25°C and 60% relative humidity. The Canton-S strain was used as the wild-type *D. melanogaster* flies. The following fly stocks, *HL09-Gal4*, *TH-Gal4*, *c739-Gal4*, *c305-Gal4*, *Y201-Gal4*, *MB247-Gal4*, *UAS-Trp*, *UAS-DopR1*, $DopR1^{attp}$, $DopR2^{attp}$, and $DopR1^{res}$ were previously described by Keleman et al. (2007) and Keleman et al. (2012). The $Orb2^{DQ}$, $Orb2^{+}$, and $Orb2^{mCPEBRBD}$ flies were generated by Krüttner et al. (2012). The *VT5526-LexA* driver and *LexAop-Trp* flies are unpublished reagents from B.J. Dickson (personal communication). The *UAS-EYFP-CaMKII3′UTR* flies were generated by Ashraf et al. (2006). All mutant flies were backcrossed to the Canton-S for four generations before being used for behavioral assays.

Behavioral Assays

Behavioral assays were executed at variable phases of the circadian clock of the flies. Courtship conditioning assays were carried out as described previously (Keleman et al., 2007; Siwicki and Ladewski, 2003). Details can be found in the Supplemental Information.

Memory-consolidation assay by dopamine feeding was performed as follows. Freshly hatched males were collected and aged individually in food chambers as for courtship conditioning. Prior to training, flies were starved on a wet filter paper for 16 hr. After short-term memory training (1 hr), at indicated time points, flies were transferred to chambers containing filter paper soaked with 80 μl of 2% sucrose solution supplemented with either dopamine, cyclohexamide, or SCH23390 as indicated (concentrations used: 20 mM dopamine, 35 mM cyclohexamide, or 1 μM SCH23390). Memory-consolidation assay by thermogenetic activation with TrpA1 was performed as follows. Freshly hatched males were collected and aged individually in food chambers at 22°C for 6 or 7 days. First, they were trained for 1 hr at 22°C, shifted to 32°C at indicated time points for 2 hr, and thereafter placed at 22°C until the test at 25°C (24 hr after training).

For silencing with Shi^{ts}, males were collected and aged as described above. They were trained for 7 hr at 22°C, shifted to 32°C at indicated time points, and thereafter placed at 22°C until the test at 25°C (24 hr after training).

TARGET experiments were conducted as described (McGuire et al., 2003). For the experiment, all flies were raised and kept at 18°C and shifted to 27°C at indicated time intervals. Test was performed at 25°C. Genotypes of the experimental flies were: *w+*: *tub-GAL80*^{ts}, *UAS-orb2A* (*UAS-orb2A*$^{\Delta Q}$); *orb2B*$^{\Delta Q}$, *MB247-Gal4* and *w+*: *tub-GAL80*^{ts}, *UAS-orb2B* (*UAS-orb2B**); *orb2*^{mCPEB2RBD}, *MB247-Gal4*.

Immunohistochemistry

Immunohistochemistry on adult brains was performed as described (Yu et al., 2010). Details on antibodies used can be found in the Supplemental Information.

Immunoprecipitation and Western Blot

IP and WB were carried out as described previously (Krüttner et al., 2012) on adult brains of *w+;+; orb2*^{GFP}, *DopR1*^{attp} and *w+;+; orb2*^{GFP}, *DopR2*^{attp} to investigate $Orb2^{GFP}$ complex formation. To determine on/off kinetics of Orb2 expression in TARGET experiment, IP and WB were performed on brain extract from *w+;tubGal80*, *UAS-orb2B;Orb2*^{mCPEBRBD}*,MB247-Gal4*. Details can be found in the Supplemental Information.

Immuno-EM

The heads of heterozygous viable *orb2*^{Orb2AGFP} and *orb2*^{Orb2BGFP} 6- or 7-day-old flies were fixed in 4% paraformalde, 0.1% glutaraldehyde, and 0.07 M phosphate buffer (pH 7.3) for 3 hr at 40°C and prepared for immuno-EM as described (Krüttner et al., 2012). Details can be found in the Supplemental Information.

Statistical Analysis

LIs were calculated using a custom MATLAB script based on the algorithm described in Kamyshev et al. (1999) and implemented in Keleman et al. (2007). Briefly, the entire set of courtship indices for both the naive and trained flies was pooled and then randomly assorted into simulated naive and trained sets of the same size as in the original data. A LI_p was calculated for each of 100,000 randomly permuted data sets, and p values were estimated as the fraction for which $LI_p > LI$ (to test H_0, $LI = 0$) or $LI_p > LI - LI_0$ (to test H_0, $LI = LI_0$). p values are for H_0: $LI = LI_1$ (permutation test) and $^{*}p < 0.05$, $^{**}p < 0.01$, and $^{***}p < 0.001$ for H_0, $LI = 0$ (permutation test). Figures in the main text show LIs ± SEM calculated using the propagation of error formula and p values calculated from mean CIs; supplemental tables show values derived from both mean ± SD and median CIs.

SUPPLEMENTAL INFORMATION

Supplemental Information includes Supplemental Experimental Procedures, two figures, and twelve tables and can be found with this article online at http://dx.doi.org/10.1016/j.celrep.2015.05.037.

AUTHOR CONTRIBUTIONS

S.K. and K.K. conceived the project and designed the experiments. S.K. and L.T. performed all the experiments with the exception of the memory-consolidation assay using virgin females as testers, silencing of the aSP13 neurons after long-term memory training, and post-acquisition inactivation of DopRs in memory consolidation and long-term memory assays, which were performed by U.D. S.K. and L.T. performed data analysis with help from U.D. K.J. helped S.K. and L.T. with behavioral experiments. B.S. performed CaMKII translation-repression assay. N.I. performed EM analysis. J.N.N. and L.G.F. made an initial observation of Orb2A localization. K.K. supervised the project and wrote the manuscript with help of B.D.M.

ACKNOWLEDGMENTS

We thank U. Heberlein, B. Dickson, and V. Jayaraman for critical comments on the manuscript; R. Fetter for supervising the EM studies; C. Kent for advice on statistics; and B. Koster, Anja de Jong, Jos Onderwater, Erik Bos, and Christina Avramut for help with the initial EM experiments. The work was supported by the grants from Austrian Science Funds to K.K. as well as FWF P21854-B09 and WWTF P21854.

Received: October 2, 2014
Revised: April 14, 2015
Accepted: May 21, 2015
Published: June 18, 2015

REFERENCES

Ashraf, S.I., McLoon, A.L., Sclarsic, S.M., and Kunes, S. (2006). Synaptic protein synthesis associated with memory is regulated by the RISC pathway in Drosophila. Cell *124*, 191–205.

Berke, J.D., Paletzki, R.F., Aronson, G.J., Hyman, S.E., and Gerfen, C.R. (1998). A complex program of striatal gene expression induced by dopaminergic stimulation. J. Neurosci. *18*, 5301–5310.

Buzsáki, G. (1998). Memory consolidation during sleep: a neurophysiological perspective. J. Sleep Res. *7* (*1*), 17–23.

Buzsáki, G., Horváth, Z., Urioste, R., Hetke, J., and Wise, K. (1992). High-frequency network oscillation in the hippocampus. Science *256*, 1025–1027.

Carr, M.F., Jadhav, S.P., and Frank, L.M. (2011). Hippocampal replay in the awake state: a potential substrate for memory consolidation and retrieval. Nat. Neurosci. *14*, 147–153.

Claridge-Chang, A., Roorda, R.D., Vrontou, E., Sjulson, L., Li, H., Hirsh, J., and Miesenböck, G. (2009). Writing memories with light-addressable reinforcement circuitry. Cell *139*, 405–415.

Coultrap, S.J., and Bayer, K.U. (2012). CaMKII regulation in information processing and storage. Trends Neurosci. *35*, 607–618.

Dickson, B.J. (2008). Wired for sex: the neurobiology of Drosophila mating decisions. Science *322*, 904–909.

Draper, I., Kurshan, P.T., McBride, E., Jackson, F.R., and Kopin, A.S. (2007). Locomotor activity is regulated by D2-like receptors in Drosophila: an anatomic and functional analysis. Dev. Neurobiol. *67*, 378–393.

Foster, D.J., and Wilson, M.A. (2006). Reverse replay of behavioural sequences in hippocampal place cells during the awake state. Nature *440*, 680–683.

Frey, U., and Morris, R.G. (1997). Synaptic tagging and long-term potentiation. Nature *385*, 533–536.

Gotzes, F., Balfanz, S., and Baumann, A. (1994). Primary structure and functional characterization of a Drosophila dopamine receptor with high homology to human D1/5 receptors. Receptors Channels *2*, 131–141.

Guo, A., Li, L., Xia, S.Z., Feng, C.H., Wolf, R., and Heisenberg, M. (1996). Conditioned visual flight orientation in Drosophila: dependence on age, practice, and diet. Learn. Mem. *3*, 49–59.

Hafer, N., Xu, S., Bhat, K.M., and Schedl, P. (2011). The Drosophila CPEB protein Orb2 has a novel expression pattern and is important for asymmetric cell division and nervous system function. Genetics *189*, 907–921.

Hearn, M.G., Ren, Y., McBride, E.W., Reveillaud, I., Beinborn, M., and Kopin, A.S. (2002). A Drosophila dopamine 2-like receptor: Molecular characterization and identification of multiple alternatively spliced variants. Proc. Natl. Acad. Sci. USA *99*, 14554–14559.

Ishimoto, H., Sakai, T., and Kitamoto, T. (2009). Ecdysone signaling regulates the formation of long-term courtship memory in adult Drosophila melanogaster. Proc. Natl. Acad. Sci. USA *106*, 6381–6386.

Jadhav, S.P., Kemere, C., German, P.W., and Frank, L.M. (2012). Awake hippocampal sharp-wave ripples support spatial memory. Science *336*, 1454–1458.

Kamyshev, N.G., Iliadi, K.G., and Bragina, J.V. (1999). Drosophila conditioned courtship: two ways of testing memory. Learn. Mem. *6*, 1–20.

Kandel, E.R. (2001). The molecular biology of memory storage: a dialogue between genes and synapses. Science *294*, 1030–1038.

Keleman, K., Krüttner, S., Alenius, M., and Dickson, B.J. (2007). Function of the Drosophila CPEB protein Orb2 in long-term courtship memory. Nat. Neurosci. *10*, 1587–1593.

Keleman, K., Vrontou, E., Krüttner, S., Yu, J.Y., Kurtovic-Kozaric, A., and Dickson, B.J. (2012). Dopamine neurons modulate pheromone responses in Drosophila courtship learning. Nature *489*, 145–149.

Kim, Y.C., Lee, H.G., Seong, C.S., and Han, K.A. (2003). Expression of a D1 dopamine receptor dDA1/DmDOP1 in the central nervous system of Drosophila melanogaster. Gene Expr. Patterns *3*, 237–245.

Krüttner, S., Stepien, B., Noordermeer, J.N., Mommaas, M.A., Mechtler, K., Dickson, B.J., and Keleman, K. (2012). Drosophila CPEB Orb2A mediates memory independent of Its RNA-binding domain. Neuron *76*, 383–395.

Lucchesi, W., Mizuno, K., and Giese, K.P. (2011). Novel insights into CaMKII function and regulation during memory formation. Brain Res. Bull. *85*, 2–8.

Majumdar, A., Cesario, W.C., White-Grindley, E., Jiang, H., Ren, F., Khan, M.R., Li, L., Choi, E.M., Kannan, K., Guo, F., et al. (2012). Critical role of amyloid-like oligomers of Drosophila Orb2 in the persistence of memory. Cell *148*, 515–529.

Mao, Z., and Davis, R.L. (2009). Eight different types of dopaminergic neurons innervate the Drosophila mushroom body neuropil: anatomical and physiological heterogeneity. Front Neural Circuits *3*, 5.

Martin, S.J., Grimwood, P.D., and Morris, R.G. (2000). Synaptic plasticity and memory: an evaluation of the hypothesis. Annu. Rev. Neurosci. *23*, 649–711.

McBride, S.M., Giuliani, G., Choi, C., Krause, P., Correale, D., Watson, K., Baker, G., and Siwicki, K.K. (1999). Mushroom body ablation impairs short-term memory and long-term memory of courtship conditioning in Drosophila melanogaster. Neuron *24*, 967–977.

McGuire, S.E., Le, P.T., Osborn, A.J., Matsumoto, K., and Davis, R.L. (2003). Spatiotemporal rescue of memory dysfunction in Drosophila. Science *302*, 1765–1768.

McGuire, S.E., Mao, Z., and Davis, R.L. (2004). Spatiotemporal gene expression targeting with the TARGET and gene-switch systems in Drosophila. Sci. STKE *2004*, pl6.

Mendez, R., and Richter, J.D. (2001). Translational control by CPEB: a means to the end. Nat. Rev. Mol. Cell Biol. *2*, 521–529.

Moncada, D., and Viola, H. (2007). Induction of long-term memory by exposure to novelty requires protein synthesis: evidence for a behavioral tagging. J. Neurosci. *27*, 7476–7481.

Musso, P.Y., Tchenio, P., and Preat, T. (2015). Delayed dopamine signaling of energy level builds appetitive long-term memory in Drosophila. Cell Rep. *10*, 1023–1031.

Nicoll, R.A., and Malenka, R.C. (1999). Expression mechanisms underlying NMDA receptor-dependent long-term potentiation. Ann. N Y Acad. Sci. *868*, 515–525.

Ofstad, T.A., Zuker, C.S., and Reiser, M.B. (2011). Visual place learning in Drosophila melanogaster. Nature *474*, 204–207.

Plaçais, P.Y., Trannoy, S., Isabel, G., Aso, Y., Siwanowicz, I., Belliart-Guérin, G., Vernier, P., Birman, S., Tanimoto, H., and Preat, T. (2012). Slow oscillations in two pairs of dopaminergic neurons gate long-term memory formation in Drosophila. Nat. Neurosci. *15*, 592–599.

Qin, H., Cressy, M., Li, W., Coravos, J.S., Izzi, S.A., and Dubnau, J. (2012). Gamma neurons mediate dopaminergic input during aversive olfactory memory formation in Drosophila. Curr. Biol. *22*, 608–614.

Quinn, W.G., Harris, W.A., and Benzer, S. (1974). Conditioned behavior in Drosophila melanogaster. Proc. Natl. Acad. Sci. USA *71*, 708–712.

Redondo, R.L., Okuno, H., Spooner, P.A., Frenguelli, B.G., Bito, H., and Morris, R.G. (2010). Synaptic tagging and capture: differential role of distinct calcium/calmodulin kinases in protein synthesis-dependent long-term potentiation. J. Neurosci. *30*, 4981–4989.

Richter, J.D. (2001). Think globally, translate locally: what mitotic spindles and neuronal synapses have in common. Proc. Natl. Acad. Sci. USA *98*, 7069–7071.

Riemensperger, T., Isabel, G., Coulom, H., Neuser, K., Seugnet, L., Kume, K., Iché-Torres, M., Cassar, M., Strauss, R., Preat, T., et al. (2011). Behavioral consequences of dopamine deficiency in the Drosophila central nervous system. Proc. Natl. Acad. Sci. USA *108*, 834–839.

Rosenzweig, M., Brennan, K.M., Tayler, T.D., Phelps, P.O., Patapoutian, A., and Garrity, P.A. (2005). The Drosophila ortholog of vertebrate TRPA1 regulates thermotaxis. Genes Dev. *19*, 419–424.

Rossato, J.I., Bevilaqua, L.R., Izquierdo, I., Medina, J.H., and Cammarota, M. (2009). Dopamine controls persistence of long-term memory storage. Science *325*, 1017–1020.

Schwaerzel, M., Monastirioti, M., Scholz, H., Friggi-Grelin, F., Birman, S., and Heisenberg, M. (2003). Dopamine and octopamine differentiate between aversive and appetitive olfactory memories in Drosophila. J. Neurosci. *23*, 10495–10502.

Si, K., Giustetto, M., Etkin, A., Hsu, R., Janisiewicz, A.M., Miniaci, M.C., Kim, J.H., Zhu, H., and Kandel, E.R. (2003a). A neuronal isoform of CPEB regulates local protein synthesis and stabilizes synapse-specific long-term facilitation in aplysia. Cell *115*, 893–904.

Si, K., Lindquist, S., and Kandel, E.R. (2003b). A neuronal isoform of the aplysia CPEB has prion-like properties. Cell *115*, 879–891.

Siegel, R.W., and Hall, J.C. (1979). Conditioned responses in courtship behavior of normal and mutant Drosophila. Proc. Natl. Acad. Sci. USA *76*, 3430–3434.

Siwicki, K.K., and Ladewski, L. (2003). Associative learning and memory in Drosophila: beyond olfactory conditioning. Behav. Processes *64*, 225–238.

Smith, W.B., Starck, S.R., Roberts, R.W., and Schuman, E.M. (2005). Dopaminergic stimulation of local protein synthesis enhances surface expression of GluR1 and synaptic transmission in hippocampal neurons. Neuron *45*, 765–779.

Srivastava, D.P., Yu, E.J., Kennedy, K., Chatwin, H., Reale, V., Hamon, M., Smith, T., and Evans, P.D. (2005). Rapid, nongenomic responses to ecdysteroids and catecholamines mediated by a novel Drosophila G-protein-coupled receptor. J. Neurosci. *25*, 6145–6155.

Sutton, M.A., and Schuman, E.M. (2006). Dendritic protein synthesis, synaptic plasticity, and memory. Cell *127*, 49–58.

Tempel, B.L., Livingstone, M.S., and Quinn, W.G. (1984). Mutations in the dopa decarboxylase gene affect learning in Drosophila. Proc. Natl. Acad. Sci. USA *81*, 3577–3581.

Theis, M., Si, K., and Kandel, E.R. (2003). Two previously undescribed members of the mouse CPEB family of genes and their inducible expression in the principal cell layers of the hippocampus. Proc. Natl. Acad. Sci. USA *100*, 9602–9607.

Waddell, S. (2010). Dopamine reveals neural circuit mechanisms of fly memory. Trends Neurosci. *33*, 457–464.

Wang, X.P., and Cooper, N.G. (2009). Characterization of the transcripts and protein isoforms for cytoplasmic polyadenylation element binding protein-3 (CPEB3) in the mouse retina. BMC Mol. Biol. *10*, 109.

Wang, H., Hu, Y., and Tsien, J.Z. (2006). Molecular and systems mechanisms of memory consolidation and storage. Prog. Neurobiol. *79*, 123–135.

White-Grindley, E., Li, L., Mohammad Khan, R., Ren, F., Saraf, A., Florens, L., and Si, K. (2014). Contribution of Orb2A stability in regulated amyloid-like oligomerization of Drosophila Orb2. PLoS Biol. *12*, e1001786.

Wilson, M.A., and McNaughton, B.L. (1994). Reactivation of hippocampal ensemble memories during sleep. Science *265*, 676–679.

Wise, R.A. (2004). Dopamine, learning and motivation. Nat. Rev. Neurosci. *5*, 483–494.

Wu, L., Wells, D., Tay, J., Mendis, D., Abbott, M.A., Barnitt, A., Quinlan, E., Heynen, A., Fallon, J.R., and Richter, J.D. (1998). CPEB-mediated cytoplasmic polyadenylation and the regulation of experience-dependent translation of alpha-CaMKII mRNA at synapses. Neuron *21*, 1129–1139.

Wustmann, G., Rein, K., Wolf, R., and Heisenberg, M. (1996). A new paradigm for operant conditioning of Drosophila melanogaster. J. Comp. Physiol. A Neuroethol. Sens. Neural Behav. Physiol. *179*, 429–436.

Yu, J.Y., Kanai, M.I., Demir, E., Jefferis, G.S., and Dickson, B.J. (2010). Cellular organization of the neural circuit that drives Drosophila courtship behavior. Curr. Biol. *20*, 1602–1614.

Cell Reports
Supplemental Information

Synaptic Orb2A Bridges Memory Acquisition and Late Memory Consolidation in *Drosophila*

Sebastian Krüttner, Lisa Traunmüller, Ugur Dag, Katharina Jandrasits, Barbara Stepien, Nirmala Iyer, Lee G. Fradkin, Jasprina N. Noordermeer, Brett D. Mensh, and Krystyna Keleman

Supplemental Information

Figure S1 VT005526-LexA drives expression exclusively in the aSP13 dopaminergic neurons (Figure 2B and 2C)

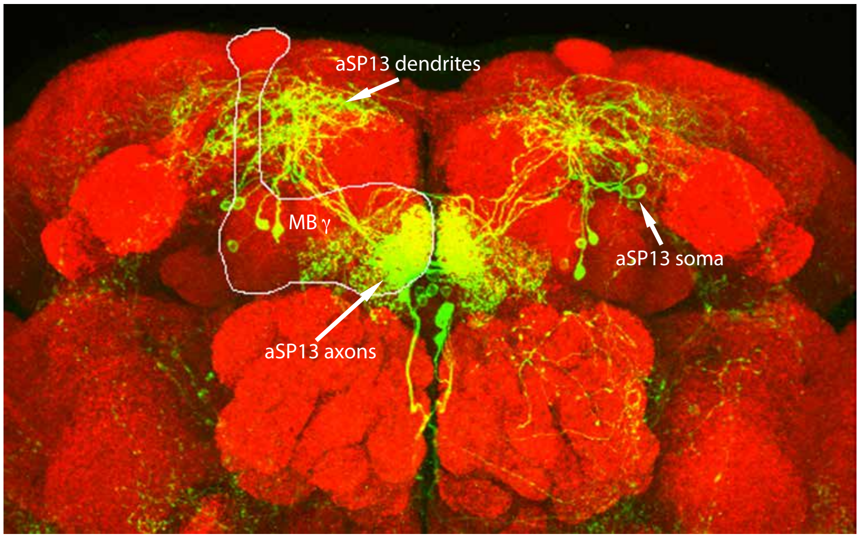

Expression pattern of the VT005526-LexA line used for the activation and silencing experiments with LexAop-TrpA1 and LexAop-shi^{ts} respectively, shown in the Figure 2B and 2C. There are typically 2-6 aSP13 neurons per hemisphere. The presynaptic termini of aSP13 neurons (axons) are located at the tip to the MB γ lobe. The postsynaptic termini (dendrites) are located in the medial protocerebrum.

Figure S2 On/Off kinetics of Orb2 in TARGET experiment (Figure 6)

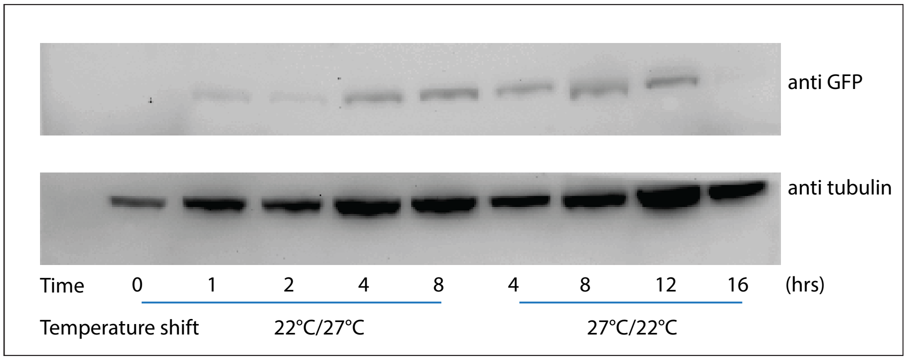

Head extracts from *w+;tubGal80ts, UAS-orb2BGFP;orb2*^{mCPEBRBD} adult flies were analyzed by IP and WB with Abs against the GFP tag at indicated time points after temperature shift either from 22°C to 27°C or back to 22°C after 7 hour induction at 27°C.

Table S1 Post-learning global activation of dopamine pathways (Figure 1)

	Genotype	Test	DA	Train	*n*	CI (%) mean±sd median	10%-ile 90%-ile	LI (%) mean±sem median	*P* *LI=0*	*P* $LI_n=LI_4$
1	*Canton-S*	20 min	-	-	57	52.3±26.3<br>50.0	15.00<br>90.00			
2	*Canton-S*	20 min	-	+	57	13.9±18.9<br>5.0	0.00<br>31.00	73.5±5.0<br>90.0	0.000<br>0.000	0.000<br>0.000
3	*Canton-S*	24 hrs	-	-	54	53.4±25.4<br>60.0	12.50<br>85.00			
4	*Canton-S*	24 hrs	-	+	61	51.1±25.3<br>55.0	15.00<br>85.00	4.4±8.7<br>8.3	0.319<br>0.473	
5	*Canton-S*	24 hrs	-	-	54	53.4±25.4<br>60.0	12.50<br>85.00			
6	*Canton-S*	24 hrs	+	-	66	57.1±29.7<br>65.0	13.50<br>91.50	-7.1±9.7<br>-8.3	0.770<br>0.878	0.386<br>0.486
7	*Canton-S*	24 hrs	+	-	66	57.2±29.7<br>65.0	13.50<br>91.50			
8	*Canton-S*	24 hrs	+	+	67	30.1±24.8<br>25.0	0.00<br>66.00	47.3±6.3<br>61.5	0.000<br>0.000	0.000<br>0.002
9	*Canton-S*	24 hrs<br>(w/ virgin)	+	-	45	79.3±28.4<br>90.0	31.00<br>100			
10	*Canton-S*)	24 hrs<br>(w/ virgin)	+	+	57	66.1±32.5<br>75.00	5.00<br>100	16.6±7.0<br>16.7	0.017<br>0.018	0.304<br>0.512

Courtship indices of the *n* Canton-S males fed with sucrose only (DA-) or supplemented with dopamine (DA+) after being trained with a mated female for 1 hr (Train +) or staying alone (Train-) as indicated in Fig. 1, and tested in single-pair assays with mated or virgin females when indicated. *P* values determined by permutation test for the null hypothesis that learning equals 0 (H_0: LI = 0) or for the null hypothesis that specific experimental and control males learn equally well (H_0: $LI_n = LI_c$)

Table S2 Post-learning thermogenetic activation of PAM-DA neurons (Figure 2A)

	Genotype	Time at 32°C	Train	*n*	CI (%) mean±sd median	10%-ile 90%-ile	LI (%) mean±sem median	*P* *LI=0*	*P* $LI_n=LI_{18}$
1	*HL09-Gal4, UAS-TrpA1*, 32°C	2-4	-	34	82.8±29.5<br>100.0	20.00<br>100			
2	*HL09-Gal4, UAS-TrpA1*, 32°C	2-4	+	36	71.6±34.3<br>90.0	8.50<br>100	13.5±5.0<br>10.0	0.067<br>0.131	0.091<br>0.028
3	*HL09-Gal4, UAS-TrpA1*, 32°C	4-6	-	34	82.5±27.5<br>95.0	29.00<br>100			
4	*HL09-Gal4, UAS-TrpA1*, 32°C	4-6	+	36	71.4±30.1<br>75.0	25.00<br>100	13.5±8.0<br>21.05	0.052<br>0.064	0.073<br>0.028
5	*HL09-Gal4, UAS-TrpA1*, 32°C	6-8	-	36	76.0±24.0<br>80.0	33.50<br>100			
6	*HL09-Gal4, UAS-TrpA1*, 32°C	6-8	+	34	65.7±30.2<br>70.0	25.00<br>100	13.5±8.2<br>12.5	0.061<br>0.244	0.075<br>0.120
7	*HL09-Gal4, UAS-TrpA1*, 32°C	8-10	-	35	83.1±21.9<br>95.0	52.50<br>100			
8	*HL09-Gal4, UAS-TrpA1*, 32°C	8-10	+	36	44.2±33.5<br>40.0	5.00<br>97.00	46.9±7.2<br>57.9	0.000<br>0.000	0.000<br>0.000
9	*HL09-Gal4, UAS-TrpA1*, 32°C	10-12	-	34	58.5±29.6<br>55.0	22.50<br>100			
10	*HL09-Gal4, UAS-TrpA1*, 32°C	10-12	+	34	64.7±36.0<br>80.0	7.50<br>100	-10.6±14.2<br>-45.4	0.772<br>0.937	0.922<br>0.680

11	*HL09-Gal4, UAS-TrpA1*, 32°C	12-14	-	36	59.3±27.9<br>60.00	23.50<br>96.50			
12	*HL09-Gal4, UAS-TrpA1*, 32°C	12-14	+	36	59.0±33.2<br>55.00	13.50<br>100	0.7±5.0<br>8.3	0.485<br>0.434	0.478<br>0.491
13	*HL09-Gal4, UAS-TrpA1*, 32°C	14-16	-	33	87.9±20.5<br>95.00	67.00<br>100			
14	*HL09-Gal4, UAS-TrpA1*, 32°C	14-16	+	36	80.7±30.4<br>95.0	15.50<br>100	8.2±6.9<br>0.0	0.134<br>0.964	0.141<br>0.009
15	*HL09-Gal4, UAS-TrpA1*, 32°C	16-18	-	33	92.6±13.1<br>95.0	77.00<br>100			
16	*HL09-Gal4, UAS-TrpA1*, 32°C	16-18	+	36	87.9±22.2<br>95.0	61.00<br>100	5.0±4.6<br>0.0	0.149<br>0.970	0.166<br>0.001
17	*HL09-Gal4, UAS-TrpA1*, 22°C	-	-	30	66.7±34.7<br>75.0	5.50<br>100			
18	*HL09-Gal4, UAS-TrpA1*, 22°C	-	+	34	75.0±33.2<br>95.0	10.00<br>100	-12.5±13.7<br>-26.6	0.835<br>0.877	
19	*UAS-TRPA1/+*, 32°C	8-10	-	35	54.9±33.1<br>55.0	10.00<br>95.00			
20	*UAS-TRPA1/+*, 32°C	8-10	+	34	55.0±30.2<br>55.0	10.00<br>92.50	-0.2±13.9<br>0.0	0.511<br>0.636	0.533<br>0.572
21	*HL09-Gal4/+*, 32°C	8-10	-	35	50.9±29.9<br>40.0	15.00<br>100			
22	*HL09-Gal4/+*, 32°C	8-10	+	35	44.3±27.6<br>35.0	15.00<br>92.00	12.9±5.0<br>12.5	0.169<br>0.242	0.208<br>0.433
23	*HL09-Gal4, UAS-TrpA1*, 32°C<br>+PSI	8-10	-	36	63.9±34.4<br>70.00	15.00<br>100			
24	*HL09-Gal4, UAS-TrpA1*, 32°C<br>+PSI	8-10	+	34	66.8±29.1<br>70.0	17.50<br>100	-4.5±12.2<br>0.0	0.642<br>0.657	0.665<br>0.573

Courtship indices of males of the indicated genotypes, retained at 22°C or warmed to 32°C for 2 hrs at the time points according to Fig. 2A after either 1 hour training with a mated female (Train+) or staying alone (Train-) as indicated above and tested in single-pair assays with mated females. *P* values determined by permutation test for the null hypothesis that learning equals 0 (H_0: LI = 0) or for the null hypothesis that specific experimental and control males learn equally well (H_0: $LI_n = LI_c$)

Table S3 Post-learning thermogenetic activation of aSP13-DA neurons (Figure 2B)

	Genotype	Time at 32°C	Train	n	CI (%)<br>mean±sd<br>median	10%-ile<br>90%-ile	LI (%)<br>mean±sem<br>median	P LI=0	P LI_n=LI_c
1	*TH-Gal4, FF, UAS-TrpA1*, 32°C	8-10	-	36	67.4±27.2<br>77.5	20.00<br>96.50			
2	*TH-Gal4, FF, UAS-TrpA1*, 32°C	8-10	+	36	45.8±28.1<br>40.0	8.50<br>90.00	32.0±5.0<br>46.7	0.001<br>0.006	0.000<br>0.097
3	*VT005526-LexA, LexAop-TrpA1*, 32°C	8-10	-	28	79.8±18.4<br>85.0	48.00<br>100			
4	*VT005526-LexA, LexAop-TrpA1*, 32°C	8-10	+	34	60.2±35.0<br>50.00	7.50<br>100	24.6±7.6<br>41.2	0.005<br>0.001	0.006<br>0.003
5	*VT005526-LexA, LexAop-TrpA1*, 22°C	-	-	34	60.9±33.9<br>65.0	12.50<br>100			
6	*VT005526-LexA, LexAop-TrpA1*, 22°C	-	+	34	71.0±25.1<br>77.5	27.50<br>100	-16.7±13.2<br>-25.0	0.915<br>0.862	
7	*VT005526-LexA/+*, 32°C	8-10	-	17	82.9±13.4<br>85.0	64.00<br>105			
8	*VT005526-LexA/+*, 32°C	8-10	+	16	80.3±20.1<br>87.5	46.00<br>100	3.2±7.1<br>0.0	0.356<br>0.891	0.272<br>0.269
9	*TH-Gal4*, 32°C	8-10	-	36	55.0±23.1<br>45.0	30.00<br>95.00			
10	*TH-Gal4*, 32°C	8-10	+	36	60.6±22.6	25.00	-10.1±10.3	0.857	0.771

					57.5	90.00	-22.2	0.906	0.407
11	*TH-Gal4, FF, UAS-TrpA1*, 22°C	-	-	33	63.9±25.1<br>75.0	25.00<br>95.00			
12	*TH-Gal4, FF, UAS-TrpA1*, 22°C	-	+	29	73.1±19.1<br>75.0	45.00<br>100	-14.3±5.0<br>0.0	0.944<br>0.769	

Courtship indices of males of the indicated genotypes, retained at 22°C or warmed to 32°C for 2 hrs according to Fig. 2B after either 1 hour training with a mated female (Train+) or staying alone (Train-) as indicated above and tested in single-pair assays with mated females. *P* values determined by permutation test for the null hypothesis that learning equals 0 (H_0: LI = 0) or for the null hypothesis that specific experimental and control males learn equally well (H_0: $LI_n = LI_c$)

Table S4 Post-learning thermogenetic silencing of aSP13-DA neurons (Figure 2C)

	Genotype	Train	*n*	CI (%)<br>mean±sd<br>median	10%-ile<br>90%-ile	LI (%)<br>mean±sem<br>median	*P*<br>*LI=0*	*P* LI_n=LI_6
1	*VT005526-LexA, LexAop-shi^{ts}*, 22°C	-	33	88.3±18.0<br>95.0	62.00<br>100			
2	*VT005526-LexA, LexAop-shi^{ts}*, 22°C	+	36	58.2±26.5<br>62.5	12.00<br>91.50	34.1±5.0<br>36.8	0.000<br>0.000	
3	*VT005526-LexA, LexAop-shi^{ts}*, 32°C	-	36	92.5±18.5<br>100	76.00<br>100			
4	*VT005526-LexA, LexAop-shi^{ts}*, 32°C	+	33	85.0±17.5<br>90.0	60.00<br>100	8.1±4.5<br>10.0	0.052<br>0.023	0.001<br>0.001
5	*LexAop-shi^{ts}*, 32°C	-	35	68.7±25.3<br>75.0	33.00<br>95.00			
6	*LexAop-shi^{ts}*, 32°C	+	33	46.7±26.9<br>45.0	5.00<br>85.00	32.1±8.0<br>40.0	0.001<br>0.001	0.831<br>0.883
7	*VT005526-LexA*, 32°C	-	29	60.9±17.5<br>65.0	35.00<br>85.00			
8	*VT005526-LexA*, 32°C	+	33	41.9±23.4<br>45.0	4.00<br>73.00	31.1±7.6<br>30.8	0.000<br>0.009	0.767<br>0.718

Courtship indices of males of the indicated genotypes, retained at 22°C or warmed to 32°C according to Fig. 2C after 7 hour training with a mated female (Train+) or staying alone (Train-) as indicated above and tested in single-pair assays with mated females. *P* values determined by permutation test for the null hypothesis that learning equals 0 (H_0: LI = 0) or for the null hypothesis that specific experimental and control males learn equally well (H_0: $LI_n = LI_c$)

Table S5 Post-acquisition inactivation of DopR1 after training for LTM (Figure 3A)

	Genotype	Train	n	CI (%)<br>mean±sd<br>median	10%-ile<br>90%-ile	LI (%)<br>mean±sem<br>median	P LI=0	P LI_n=LI_2
1	*Canton-S*	-	59	78.7±21.5<br>85.0	45.00<br>100			
2	*Canton-S*	+	60	49.8±30.8<br>45.0	10.00<br>100	36.8±5.0<br>47.1	0.000<br>0.000	
3	*DopR1^{attp}*	-	54	78.7±22.8<br>85.0	42.50<br>100			
4	*DopR1^{attp}*	+	51	70.4±28.6<br>75.0	21.00<br>97.00	10.6±6.2<br>11.8	0.051<br>0.074	0.002<br>0.006
5	*DopR2^{attp}*	-	69	67.0±25.9<br>75.0	35.00<br>95.00			
6	*DopR2^{attp}*	+	67	41.3±32.2<br>30.0	5.00<br>91.00	38.4±6.5<br>60.0	0.000<br>0.000	0.857<br>0.381
7	*DopR1*^{res}	-	30	65.5±28.8<br>65.0	20.00<br>100			
8	*DopR1*^{res}	+	31	45.7±27.9	6.00	30.3±9.5	0.004	0.521

				45.0	89.00	30.8	0.051	0.142
9	*Canton-S*+ SCH23390	-	36	63.0±26.9<br>65.0	23.50<br>96.50			
10	*Canton-S*+ SCH23390	+	36	63.1±24.5<br>60.0	32.00<br>95.00	-0.4±9.7<br>7.7	0.519<br>0.402	0.000<br>0.003
11	$DopR2^{attp}$+ SCH23390	-	36	40.0±24.9<br>35.0	85.50<br>78.00			
12	$DopR2^{attp}$+ SCH23390	+	32	32.7±24.3<br>25.0	50.00<br>78.50	18.4±13.6<br>28.6	0.108<br>0.230	0.127<br>0.323<br>P LI_n=LI_{10}

Courtship indices of males of the indicated genotypes either trained for 7 hrs with a mated female (Train+) or remaining alone (Train-) as indicated in Fig. 3A and tested in single-pair assays with mated females. Indicated males were fed for 6 hrs with DopR1&2 antagonist, SCH23390, after 7 hrs training on water only. *P* values determined by permutation test for the null hypothesis that learning equals 0 (H_0: LI = 0) or for the null hypothesis that specific experimental and control males learn equally well (H_0: $LI_n = LI_c$)

Table S6 Post-acquisition inactivation of DopR1 (Figure 3B)

	Genotype	DA	Train	n	CI (%)<br>mean±sd<br>median	10%-ile<br>90%-ile	LI (%)<br>mean±sem<br>median	P LI=0	P LI_n=LI_2
1	*Canton-S*	+	-	72	63.4±29.4<br>75.0	16.50<br>98.50			
2	*Canton-S*	+	+	70	39.4±28.6<br>35.00	0.50<br>89.50	39.7±5.0<br>53.3	0.000<br>0.000	
3	$DopR1^{attp}$	+	-	36	70.9±27.7<br>75.0	27.50<br>100			
4	$DopR1^{attp}$	+	+	33	72.1±22.8<br>75.0	37.00<br>100	-1.6±8.7<br>0.0	0.572<br>0.632	0.000<br>0.001
5	$DopR2^{attp}$	+	-	53	58.6±27.9<br>60.0	15.00<br>95.00			
6	$DopR2^{attp}$	+	+	67	34.6±30.0<br>25.0	0.00<br>82.00	40.9±7.4<br>58.3	0.000<br>0.000	0.893<br>0.675
7	*Canton-S*	+<br>SCH23390	-	35	57.1±23.4<br>60.0	30.00<br>90.00			
8	*Canton-S*	+<br>SCH23390	+	35	58.0±23.9<br>65.0	23.00<br>87.00	-1.5±10.0<br>-8.3	0.561<br>0.762	0.001<br>0.002
9	$DopR2^{attp}$	+<br>SCH23390	-	36	44.4±31.0<br>40.0	1.50<br>85.00			
10	$DopR2^{attp}$	+<br>SCH23390	+	32	37.5±29.7<br>30.0	3.50<br>81.50	15.5±15.3<br>25.0	0.181<br>0.177	0.355<br>0.303<br>P LI_n=LI_8

Courtship indices of males of the indicated genotypes fed with sucrose supplemented with dopamine (DA+) after being starved for 16 hrs and trained with a mated female for 1 hr (Train+) or remaining alone (Train-) as indicated in Fig. 3B and tested in single-pair assays with mated females. Indicated males were fed with dopamine supplemented with DopR1&2 antagonist SCH23390. *P* values determined by permutation test for the null hypothesis that learning equals 0 (H_0: LI = 0) or for the null hypothesis that specific experimental and control males learn equally well (H_0: $LI_n = LI_c$)

Table S7 LTM rescue with DopR1 in subsets of MB neurons (Figure 3C)

	Genotype	Train	n	CI (%)<br>mean±sd<br>median	10%-ile<br>90%-ile	LI (%)<br>mean±sem<br>median	P LI=0	P LI_n=LI_2
1	*UAS-DopR1; DopR1*^{attp}	-	31	82.0±23.4<br>90.0	55.00<br>100			
2	*UAS-DopR1; DopR1*^{attp}	+	33	77.1±22.0	42.50	6.0±5.0	0.177	

				85.0	97.50	5.6	0.263	
3	*UAS-DopR1;c739-Gal4; DopR1*^{attp}	-	29	53.7±25.4<br>60.0	15.00<br>85.00			
4	*UAS-DopR1;c739-Gal4; DopR1*^{attp}	+	34	54.0±30.3<br>50.0	10.00<br>90.00	-0.6±13.0<br>16.7	0.518<br>0.402	0.644<br>0.681
5	*UAS-DopR1;305-Gal4; DopR1*^{attp}	-	20	84.5±15.7<br>90.0	60.00<br>100			
6	*UAS-DopR1;305-Gal4; DopR1*^{attp}	+	19	84.5±14.9<br>90.0	55.00<br>100	0.03±5.8<br>0.0	0.519<br>0.553	0.501<br>0.711
7	*UAS-DopR1;Y201-Gal4; DopR1*^{attp}	-	34	74.5±21.9<br>80.0	41.00<br>95.00			
8	*UAS-DopR1;Y201-Gal4; DopR1*^{attp}	+	36	44.9±26.0<br>42.5	10.00<br>80.00	39.8±6.6<br>50.0	0.000<br>0.000	0.001<br>0.009
9	*c739-Gal4; DopR1*^{attp}	-	31	89.7±14.5<br>95.0	67.00<br>100			
10	*c739-Gal4; DopR1*^{attp}	+	33	86.9±17.9<br>80.0	60.00<br>100	3.1±4.2<br>0.00	0.244<br>0.922	0.692<br>0.347
11	*305-Gal4; DopR1*^{attp}	-	32	77.8±21.0<br>75.0	45.00<br>98.50			
12	*305-Gal4; DopR1*^{attp}	+	36	65.1±24.5<br>67.5	28.50<br>100	16.3±6.6<br>23.5	0.014<br>0.004	0.261<br>0.087
13	*201-Gal4; DopR1*^{attp}	-	34	82.2±18.7<br>87.5	57.50<br>100			
14	*201-Gal4; DopR1*^{attp}	+	34	81.8±19.1<br>90.0	55.00<br>100	0.5±5.6<br>-5.9	0.484<br>0.868	0.500<br>0.339

Courtship indices of males of the indicated genotypes either trained for 7 hrs with a mated female (Train+) or remaining alone (Train-) as indicated in Fig. 3C and tested in single-pair assays with mated females. *P* values determined by permutation test for the null hypothesis that learning equals 0 (H_0: LI = 0) or for the null hypothesis that specific experimental and control males learn equally well (H_0: $LI_n = LI_c$)

Table S8 *orb2* mutant in courtship memory consolidation assay (Figure 4A)

	**Genotype**	**Train**	**DA**	**n**	**CI (%)**<br>mean±sd<br>median	**10%-ile**<br>**90%-ile**	**LI (%)**<br>mean±sem<br>median	**P LI=0**	**P**<br>LI_n=LI_2
1	*Canton-S*	-	+	36	58.5±32.1<br>55.0	10.00<br>100			
2	*Canton-S*	+	+	35	27.9±25.2<br>25.0	0.00<br>62.00	52.3±8.5<br>54.5	0.000<br>0.000	
3	*orb2*$^{orb2\Delta Q}$	-	+	36	76.0±23.0<br>80.0	33.50<br>100			
4	*orb2*$^{orb2\Delta Q}$	+	+	33	73.5±19.3<br>75.0	45.00<br>98.00	3.2±6.8<br>6.2	0.330<br>0.209	0.000<br>0.022

Courtship indices of males of the indicated genotype fed with sucrose supplemented with dopamine (+ DA) after being trained with a mated female for 1 hr (Train+) or remaining alone (Train-) as indicated in Fig. 4A and tested in single-pair assays with mated females. *P* values determined by permutation test for the null hypothesis that learning equals 0 (H_0: LI = 0) or for the null hypothesis that specific experimental and control males learn equally well (H_0: $LI_n = LI_c$)

Table S9 Quantification of the Orb2 oligomers (Figure 4C)

Genotype	***Wt (-DA)***	***DopR1***$^{att\,p}$***(-DA)***	***DopR2***^{attp} ***(-DA)***	***Wt (+DA)***	***DopR1***^{att} ***(+DA)***	***DopR2***^{attp} ***(+DA)***
Mean Intensity	358.83	316.12	229.34	6523.18	204.30	5546.77
MI_N /MI_{wt-DA}	1	0.88	0.64	18.18	0.79	15.46

WB signal corresponding to the Orb2-GFP oligomers (Fig. 4B) has been quantified using Fiji-ImageJ (Fig. 4C). Mean intensity was normalized to the wild type not treated with dopamine (wt - DA).

Table S10 Temporal rescue of LTM with Orb2A and Orb2B isoforms in MB (Figure 6)

	Genotype	*n*	CI naïve (%)<br>mean±sd<br>median	10%-ile<br>90%-ile	*n*	CI exp (%)<br>mean±sd<br>median	10%-ile<br>90%-ile	LI (%)<br>mean±sem<br>median	*P*<br>*LI=0*	*P*<br>$LI_{wt}=LI_*$
1	*TubG80^{ts};orb2$^{\Delta Q\Delta A}$;UAS-Orb2A;MB247-Gal4*	34	26.8±29.5<br>10.0	0<br>77.50	36	24.9±21.8<br>15.0	0.00<br>58.00	7.1±5.0<br>-50.0	0.382<br>0.783	
1	*TubG80ts; orb2$^{\Delta Q\Delta A}$;UAS-Orb2AΔQ;MB247-Gal4*	34	20.4±19.2<br>15.0	0<br>46.5	36	23.1±19.5<br>20.0	0.00<br>46.5	-12.8±2.3<br>-33.3	0.716<br>0.852	0.545<br>0.931
2	*TubG80ts; orb2$^{\Delta Q\Delta A}$;UAS-Orb2A;MB247-Gal4*	35	71.4±17.5<br>75.0	45.00<br>90.00	36	48.6±28.9<br>45.0	3.50<br>81.50	31.9±7.2<br>40.0	0.000<br>0.000	
2	*TubG80ts; orb2$^{\Delta Q\Delta A}$;UAS-Orb2AΔQ;MB247- Gal4*	33	62.1±27.6<br>70.00	50.00<br>95.00	43	57.2±28.2<br>65.0	10.00<br>95.00	7.9±9.9<br>7.1	0.222<br>0.499	0.039<br>0.109
3	*TubG80ts; orb2$^{\Delta Q\Delta A}$;UAS-Orb2A;MB247-Gal4*	33	48.3±27.4<br>45.00	12.00<br>91.00	34	31.6±28.8<br>25.0	0.00<br>80.00	34.6±12.3<br>44.4	0.008<br>0.015	
3	*TubG80ts; orb2$^{\Delta Q\Delta A}$;UAS-Orb2AΔQ;MB247-Gal4*	39	54.1±26.2<br>60.0	9.00<br>85.55	31	54.2±26.1<br>60.0	10.01<br>0.00	-0.2±5.0<br>0.0	0.498<br>0.571	0.046<br>0.049
4	*TubG80ts; orb2$^{\Delta Q\Delta A}$;UAS-Orb2A;MB247-Gal4*	31	68.4±28.8<br>75.0	10.00<br>95.00	36	72.5±18.8<br>75.0	42.00<br>95.00	-6.0±9.1<br>0.0	0.760<br>0.843	
4	*TubG80ts; orb2$^{\Delta Q\Delta A}$;UAS-Orb2AΔQ;MB247-Gal4*	36	67.6±27.5<br>75.0	20.50<br>95.00	34	66.9±24.5<br>70.0	27.50<br>95.00	1.1±9.0<br>6.7	0.463<br>0.421	0.578<br>0.744
5	*TubG80ts; orb2$^{\Delta Q\Delta A}$;UAS-Orb2A;MB247-Gal4*	33	74.4±20.9<br>85.0	35.00<br>95.00	36	66.1±23.0<br>70.0	28.50<br>91.50	11.1±6.8<br>17.6	0.062<br>0.026	
5	*TubG80ts; orb2$^{\Delta Q\Delta A}$;UAS-Orb2AΔQ;MB247-Gal4*	34	77.7±22.8<br>85.0	57.50<br>100	34	75.1±27.9<br>85.0	27.50<br>100	3.2±7.6<br>0.0	0.343<br>0.674	0.442<br>0.102
1’	*TubG80ts;orb2^{mCPEB2RBD};UAS-Orb2B;MB247-Gal4*	32	57.0±30.7<br>60.0	15.00<br>95.00	36	52.9±28.5<br>45.0	13.50<br>95.00	7.2±5.0<br>25.0	0.285<br>0.245	
1’	*TubG80ts;orb2^{mCPEB2RBD};UAS-Orb2BRBD*;MB247-Gal4*	18	54.2±25.8<br>50.0	19.50<br>95.50	18	45.6±17.8<br>45.0	9.50<br>76.00	15.9±13.0<br>10.0	0.136<br>0.374	0.650<br>0.645
2’	*TubG80ts;orb2^{mCPEB2RBD};UAS-Orb2B;MB247-Gal4*	35	79.9±21.9<br>85.0	45.00<br>100	34	57.2±27.1<br>65.0	7.50<br>85.00	28.4±6.8<br>23.52	0.000<br>0.000	
2’	*TubG80ts;orb2^{mCPEB2RBD};UAS-Orb2BRBD*;MB247-Gal4*	33	81.52±15.93<br>85.0	62.50<br>97.50	34	75.6±21.6<br>80.0	45.00<br>97.50	7.27±5.5<br>5.9	0.111<br>0.890	0.021<br>0.033
3’	*TubG80ts;orb2^{mCPEB2RBD};UAS-Orb2B;MB247-Gal4*	35	66.6±22.5<br>70.0	30.00<br>92.00	34	62.7±26.9<br>65.0	23.00<br>95.00	5.9±8.6<br>7.1	0.263<br>0.550	
3’	*TubG80ts;orb2^{mCPEB2RBD};UAS-Orb2BRBD*;MB247-Gal4*	26	52.9±33.0<br>60.0	3.00<br>95.00	27	46.9±33.7<br>40.0	5.00<br>91.00	11.4±5.0<br>33.3	0.273<br>0.134	0.747<br>0.268
4’	*TubG80ts;orb2^{mCPEB2RBD};UAS-Orb2B;MB247- Gal4*	36	69.7±26.9<br>80.0	27.00<br>95.00	36	67.8±21.6<br>65.0	33.50<br>95.00	2.8±8.1<br>18.8	0.365<br>0.096	
4’	*TubG80ts;orb2^{mCPEB2RBD};UAS-Orb2BRBD*;MB247-Gal4*	32	63.9±25.4<br>75.0	19.50<br>93.50	36	61.3±26.6<br>65.0	18.50<br>95.00	4.2±9.6<br>13.3	0.336<br>0.195	0.911<br>0.721
5’	*TubG80ts;orb2^{mCPEB2RBD};UAS-Orb2B;MB247-Gal4*	36	79.0±23.3<br>85.0	41.00<br>100	36	57.5±28.1<br>65.0	5.00<br>85.00	27.2±7.1<br>23.5	0.000<br>0.007	
5’	*TubG80ts;orb2^{mCPEB2RBD};UAS-Orb2BRBD*;MB247-Gal4*	25	79.6±24.2<br>90.0	38.00<br>97.00	32	73.3±26.8<br>85.0	31.00<br>100	7.9±8.1<br>5.6	0.188<br>0.277	0.074<br>0.266

Courtship indices of males of the indicated genotypes either trained for 7 hrs with a mated female (CI exp) or remaining alone (CI naive), treated as indicated in Fig. 6 and tested in single-pair assays with mated females. *P* values determined by permutation test for the null hypothesis that learning equals 0 (H_0: LI = 0) or for the null hypothesis that rescue flies with the wild type isoform learns equally well as rescue flies with the mutated isoform in the same conditions (H_0: LI_n = LI_*)

Table S11 Orb2 regulates translation of CaMKII (Figure 7A)

3'UTR	Npc2a-3'UTR-RA (control)			CaMKII-3'UTR-RH		
**Genotype**	**Orb2 wt**	**Orb2RRM***	**Ratio wt/RRM***	**Orb2 wt**	**Orb2RRM***	**Ratio wt/RRM***
FLuc/Rluc	16.183	14.680	1.1023	11.995	12.896	0.931
	16.272	15.319	1.0622	12.388	12.413	0.997
	45.179	43.740	1.0329	23.614	26.492	0.891
	42.370	44.147	0.9597	46.313	52.440	0.883
	6.380	4.599	1.3992	29.167	29.608	0.985
	7.629	7.367	1.0355	30.007	33.264	0.902
				14.852	21.392	0.694
Mean			1.098			0.896
SEM			0.0630			0.0164
P (T-test)			0.0225			

Dual luciferase reporter assay is S2 cells co-expressing either Firefly luciferase tethered to the CaMKII 3'UTR or control Npc2a-3'UTR (does not contain Orb2 specific binding sequence) and Renilla luciferase tethered to the SV40 3'UTR (Fig. 7A). The values represent Firefly luciferase signal normalized to Renilla luciferase fluorescence in S2 cells expressing either Orb2 wt or Orb2 with the RBD mutated, Orb2RRM*.

Table S12 Mean intensity of the *EYFP-CaMKII-3'UTR* in the MB gamma neurons (Figure 7C)

	Genotype	DA (hrs)	n	Mean intensity	SEM	P(ftx=ft0)	P (ftx=ft24)
1	+ *CamKII 3'UTR, wt Orb2*	0	4	28.21	5.5		0.99
2	+ *CamKII 3'UTR, wt Orb2*	6	3	50.50	4.8	0.03	0.04
3	+ *CamKII 3'UTR, wt Orb2*	12	6	50.63	4.9	0.02	0.03
4	+ *CamKII 3'UTR, wt Orb2*	24	3	28.23	6.2	0.99	

	Genotype	DA (hrs)	*n*	Mean intensity	SEM	*P(*ftx=ft0)
1	+ *CamKII 3'UTR*, $Orb2^{\Delta A}$,	0	6	21.96	4.3	
2	+ *CamKII 3'UTR*, $Orb2^{\Delta A}$	6	4	29.92	6.89	0.33

Medium intensity of the fluorescence measured in the gamma lobe of the MB of the indicated genotype according to Fig. 7C. *P* values determined by 2-sided t-test for the null hypothesis that the fluorescence intensity at time xhr equals the intensity at 0hr (H_0: ftx=ft0) or 24 hrs (H_0: ftx=ft24)

Material and methods

Courtship Conditioning Paradigm

Flies were maintained on conventional cornmeal-agar medium under a 12 hrs light: dark cycle at 25°C and 60% relative humidity. Courtship assays were performed at variable circadian clock of the flies. Males were assayed for courtship conditioning as described (Siwicki and Ladewski, 2003). For training, individual males were placed in food chambers either with (trained) or without (naive) a single premated female. After training, each male was recovered, transferred to a fresh food vial and kept in isolation until testing. For long-term memory, males were trained for 6–7 hrs and tested after 24 hrs. For short-term memory, the training period was 1 hr and the test was performed after 30 min. Tests were performed in a 10 mm diameter courtship chamber and

videotaped for 10 min (JVC handycam, 30 GB HD). Videos were scored manually and blind to the genotype for CI, which is the percentage of time each male spent courting during the test. Courtship index (CI) was used to calculate the Learning Index (LI): CI_{naive}-$CI_{trained}$/CI_{naive} × 100.

Immunohistochemistry

Immunohistochemistry on adult brains was performed as described (Yu et al., 2010). Fly brains were dissected (between 5 to 8 days after eclosion) in PBS and fixed using 4% paraformaldehyde in PBST (PBS with 0.3% Triton X-100) for 20 min at 24°C. After washing in PBST, the tissue was blocked in 5% normal goat serum in PBST for at least 2 hrs. The primary antibody and secondary antibody were incubated for 48 hrs at 4°C. The brains were washed with PBST 3 × 10 min and then overnight at 4°C between the primary and secondary antibody incubations. After the secondary antibody incubation, samples were washed 3 × 10 min and overnight at 4°C before mounting in Vectashield (VectorLabs). Antibodies used: rabbit polyclonal anti-GFP (1:5,000, Torri Pines); secondary Alexa-488 antibodies (1:1,000, Invitrogen).

Confocal Microscopy

For imaging and measurement of the fluorescence intensity of the EYFP+/- CaMKII-3'UTR, the fly brains immunostained as described above, were scanned using a Zeiss LSM 710 with a Zeiss Multi Immersion Plan NeoFluar 63× objective. Scanning parameters were set to image the entire mushroom body. Images were taken at 785 × 785 pixels. Images were processed in Imaris for fluorescence quantification. Briefly a cuboid of similar size was set as surface into each MB gamma lobe and the mean YFP fluorescence quantified.

Immunoprecipitation and Western Blot

Adult heads of the indicated genotype were lysed in homogenization buffer (PBS,150mM NaCl, 0.1mM CaCl2, 3mM MgCl2, 5% Glycerol, 1mM DTT, 0.1% TritonX100, 0.1% NP40, EDTA free protease inhibitor cocktail from Roche). The lysate was cleared by centrifugation prior to incubation with Chromotek GFPtrap beads (according to the manufacturer protocol). The proteins were transferred to a PVDF membrane (Millipore) overnight in the cold room at 35mv. Membrane was blocked in 5% milk prior to incubation for 1 hr with a primary antibody. After 3 washes in PBST (PBS+ 0,05%Tween20) membrane was incubated for 1 hr in a secondary antibody. The membrane was developed using SuperSignal West Femto Maximum Sensitivity Substrate (ThermoScientific). Antibodies used: anti-GFP (Abcam 6556 rabbit polyclonal, 1:2,000).

Immuno-EM on adult brains

The brains of 6-7 day old adult flies were dissected in cold fly saline and fixed for 3 hours on ice with 0.1% glutaraldehyde/4% formaldehyde in 0.07M sodium phosphate buffer, pH 7.4, rinsed with 0.1 M phosphate buffer containing 0.1% saponin, and incubated overnight with an HRP-conjugated rabbit anti-GFP polyclonal antibody (Life Technologies, A10260, anti-GFP, rabbit IgG fraction, horseradish peroxidase conjugate) at 1:200 dilution in 0.1 M phosphate buffer containing 5% normal goat serum/1% BSA at 4° C. The brains were then rinsed with 0.1 M phosphate/0.1% saponin buffer and reacted with 0.5 mg/ml DAB in 0.1 M phosphate buffer containing 0.1% saponin for 45 minutes following the addition of 10 μl 0.03% H_2O_2. The brains were then rinsed with 0.1 M Na-cacodylate buffer, followed by OTOT enhancement (10 min cycles of 0.01% OsO4 in 0.1M Na-cacodylate buffer followed by 0.1% thiocarbohydrazid1M Na-

cacodylate buffer with 0.1M Na-cacodylate buffer rinses between steps), and a final 1% OsO_4 in 0.1 M Na-cacodylate buffer step for 1 hour at room temperature. Following osmication, the samples were rinsed with 0.1 M Na-cacodylate buffer, water and then dehydrated in ethanol followed by propylene oxide and embedded in Eponate 12 resin (Ted Pella, Redding, CA). Embedded brains were imaged using a Zeiss Versa 510 X-ray microscope operated at 40kV and 0.7 mm/pixel resolution. The computed tomograms were used to provide coordinates of the cell bodies of the Kenyon cells and gamma lobes of the mushroom body in each sample. A Leica Ultracut 6 ultramicrotome was used to cut 90 nm sections at the level of the Kenyon cells somata and gamma lobes of the mushroom body. Unstained sections were imaged with an FEI Spirit BioTWIN TEM operated at 80kV.

CaMKII translation suppression assay

Luciferase reporter assay was done essentially as described (Mastushita-Sakai et al., 2010). In short, Orb2 plasmids were prepared by amplifying Orb2 CDS using primers containing attB sites. For Orb2 RRM1&2* site directed mutagenesis was used. All primers are listed below. Products were cloned into pDONR221 and recombined to obtain pAWM-Orb2B WT or RRM1&2* vectors. To create control (*Renilla*) and test (Firefly) luciferase expressing constructs pAMW (The Drosophila Gateway Vector Collection) was cut with BamHI (Fermentas) and the backbone fragment was ligated with MCS (multiple cloning site). Next, the Gateway expression cassette was PCR amplified from pAMW with casAWMf/casAWMr primers and cloned into the pAMW-MCS linearised with NheI/Asp718 (Roche) to obtain pAMW-cassette. For pAMW-Fluc-cassette destination vector the Firefly luciferase PCR product obtained by amplifying pAC-Fluc-6xBS with Flucf/Flucr primers was digested with SpeI/NheI and cloned into NheI cut pAMW-cassette vector. For pAMW-Rluc-cassette-polyA first the polyA signal sequence was amplified from pAMW with polAf/polAr primers and cloned with Asp718/XhoI. Subsequently, *Renilla* luciferase was PCR amplified from pAC-Rluc with Rlucf/Rlucr primers and cloned SpeI/NheI into NheI cut vector. To create final pAMW-Rluc-SV403'UTR-polyA vector SV40 3'UTR was PCR amplified from pAMW with SV403UTRf/SV403UTRr primers and cloned first into pDONR221 and then recombined with pAMW-Rluc-cassette-polyA. CaMKII 3'UTR and Npc2a-RA 3'UTR were PCR amplified using the following primers containing attB sites. PCR product was sub-cloned into pDONR221 vector using Gateway technology (Life Technologies). Resulting entry clones were recombined with pAMW-Fluc-cassette to obtain pAMW-Fluc-3'UTR plasmids. All constructs were confirmed by sequencing.

S2 cells were grown in semi-adhering liquid cultures at 27°C in water-jacketed incubator, with 5% CO_2 in liquid Schneider's Drosophila Medium (Invitrogen) supplemented with 10% fetal calf serum and PenStrep (Invitrogen) without agitation. S2 cells were split 1:10, grown overnight and diluted in Schneider's Drosophila Medium to 1mln/ml. Cells were transferred to 96-well culture plates 100µl per well. 120ng of DNA was used per transfection containing 10ng of pAMW-Rluc-SV40-polyA, 10ng of pAMW-Fluc-3'UTR reporter plasmid and 5ng pAWM-protein filled up to final DNA amount by inert bacterial plasmid pGEX-2T (GE Healthcare). Cells were transfected by adding 4.7µl of DNA/FuGENE (Promega FuGENE® HD Transfection Reagent) mix per 100ul cells and pipetting up and down. After 48h cells were transferred to deep well plates in 600µl 1xPBS, harvested by spinning 5 minutes at 800g, washed twice with 400µl 1xPBS and lysed in 30µl 1xPassive Lysis Buffer (Dual-Luciferase Reporter Assay System, Promega). For dual luciferase assay 10µl of lysate was pipetted onto 96-well plate and luciferase signals were measured in Synergy Plate Reader (BioTek) by adding 20µl Luciferase Assay Substrate in Luciferase Assay Buffer II, shaking, incubating 2 minutes and measure and 20µl Stop & Glo Substrate in Stop & Glo Buffer, shaking, incubating 2 minutes and measuring. Firefly luciferase signal was normalised to *Renilla* luciferase and ratio between signal from cells expressing Orb2B WT to Orb2B RRM1&2* mutant protein was calculated from the means for 3 replicates from the

same transfection. Each reaction was repeated in at least 6 independent experiments. Mean ratios between WT and mutant protein signals were calculated and compared to negative control (Npc2a-RA 3'UTR) using unpaired Student *t* test.

Mastushita-Sakai, T., White-Grindley, E., Samuelson, J., Seidel, C., and Si, K. (2010). Drosophila Orb2 targets genes involved in neuronal growth, synapse formation, and protein turnover. Proc Natl Acad Sci U S A *107*, 11987-11992.
Siwicki, K.K., and Ladewski, L. (2003). Associative learning and memory in Drosophila: beyond olfactory conditioning. Behav Processes *64*, 225-238.
Yu, J.Y., Kanai, M.I., Demir, E., Jefferis, G.S., and Dickson, B.J. (2010). Cellular organization of the neural circuit that drives Drosophila courtship behavior. Curr Biol *20*, 1602-1614.
